# Aryl Hydrocarbon Receptor in Health and Disease

**DOI:** 10.1002/mco2.70426

**Published:** 2025-10-17

**Authors:** Haonan Li, Yufeng Fan, Jizheng Liu, Shumin Dong, Bin Wen, Yunfei Zhang, Xiaocui Wang, Xuemei Duan, Ying Hu, Ze Yan, Huifeng Shang, Yukai Jing

**Affiliations:** ^1^ Department of Clinical Laboratory Third Hospital of Shanxi Medical University Shanxi Bethune Hospital Shanxi Academy of Medical Sciences Tongji Shanxi Hospital Taiyuan China; ^2^ The Second People's Hospital of Liaocheng Liaocheng China; ^3^ Academy of Medical Sciences Shanxi Medical University Taiyuan China

**Keywords:** AhR, immune cells, signaling pathways, treatment strategies

## Abstract

The aryl hydrocarbon receptor (AhR) functions as a ligand‐dependent transcription factor, serving as a pivotal environmental sensor that significantly influences both physiological and pathological processes. The inactivated state of AhR is present in the cell cytoplasm and transfer into the nucleus upon activation by a variety of ligands. It subsequently regulates a variety of processes including cellular metabolism, organ and tissue development, and maintenance of immune homeostasis. Despite substantial advancements over the past decade, the mechanisms by which AhR specifically regulates immune cell function in response to environmental factors and influences disease progression remain not fully elucidated. This review systematically analyzes the basic structure and major signaling pathways of AhR, its physiological functions in maintaining organismal homeostasis and its mechanism of action on various types of immune cells, and their therapeutic potential in autoimmune diseases, inflammatory disorders, tumor microenvironment, and neurodegenerative diseases. Translating immune‐metabolic reprogramming mechanisms into clinical applications represents a pivotal challenge in AhR research. And this review integrates and analyzes the great potential of AhR as a pleiotropic therapeutic target for regulating immunity and treating a series of diseases, offering actionable frameworks for future exploration.

## Graphical abstract

Based on the structure and ligands of AhR, this review introduces the AhR‐related signaling pathways and their roles in health and diseases. Agonists and antagonists of AhR as well as new strategies for treatment using the microbial–AhR axis are summarized. A prospect was made for the future use of AhR as a therapeutic target.

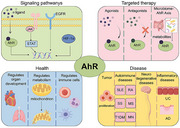

## 1 Introduction

Since the discovery of the aryl hydrocarbon receptor (AhR) as a receptor for dioxin in the 1970s, early studies have focused on its toxic metabolism [1]. In the past two decades, breakthroughs have been made in the research of AhR, which integrates signals from the environment, diet, microbiome, and endogenous metabolism and plays a role in the maintenance of immune cells and body homeostasis [2, 3]. Consequently, AhR has emerged as a pivotal regulator within the field of immune metabolism; this also establishes AhR as a target in disease intervention.

Although current studies have tentatively elucidated the mechanism of AhR regulation on immune cells, there are still some unresolved blind spots. AhR signaling pathway exhibits duality in disease pathogenesis: while its activation is essential for suppressing pathological immune hyperactivation in autoimmune disorders; paradoxically, it promotes immunosuppression within the tumor microenvironment to attenuate antitumor immunity [4, 5]. Another major challenge is how to achieve effective clinical translation and solve the problem of ligand toxicity off‐target and precise delivery to the corresponding tissues. In order to better promote the development of related fields, this review summarizes the role of AhR in health and diseases and provides a comprehensive and new overview of AhR pathway.

**FIGURE 1:**
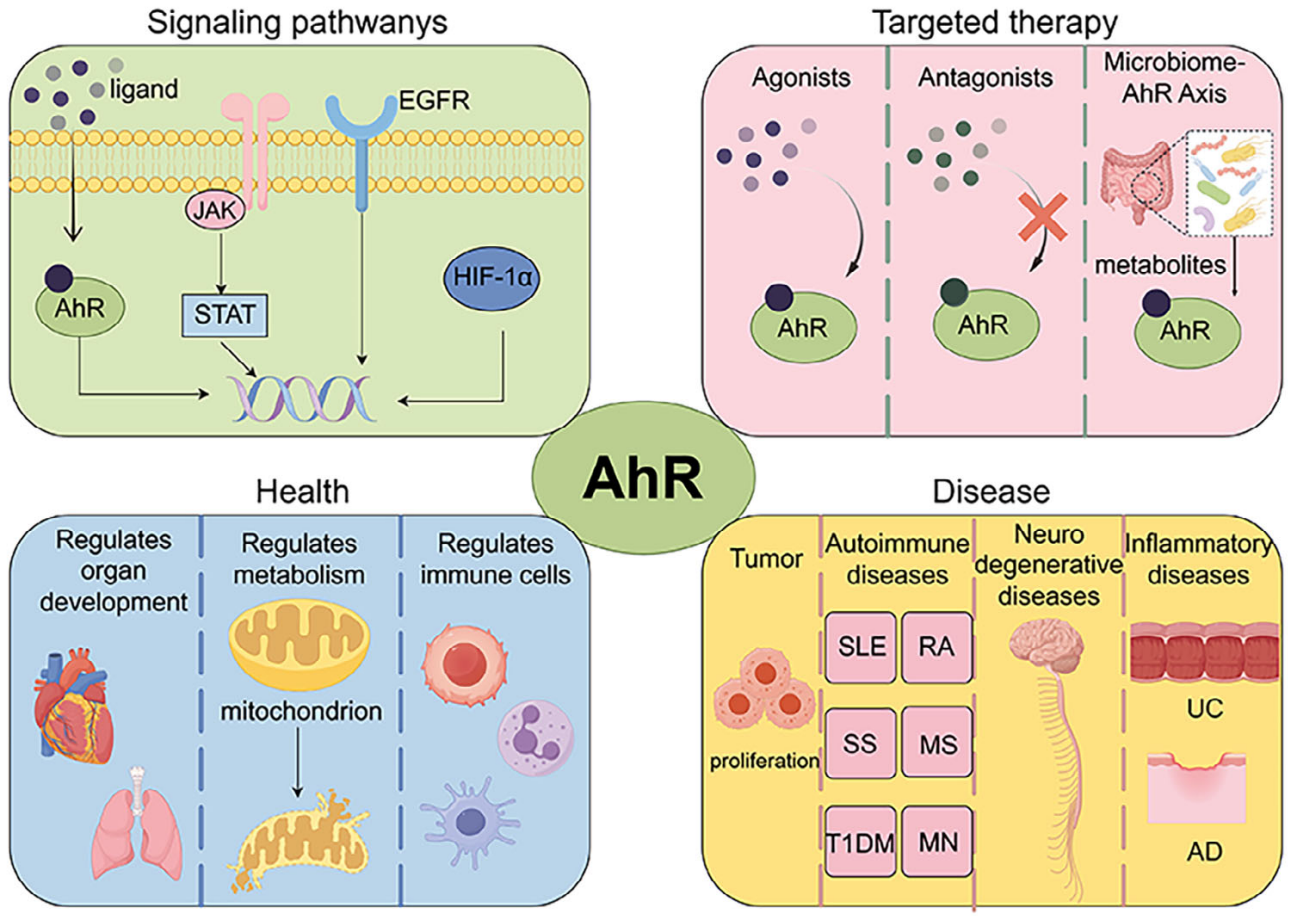
A concise figure summarizes the main content of this review. Based on the structure and ligands of the aryl hydrocarbon receptor (AhR), this review introduces the AhR‐related signaling pathways and their roles in health and diseases. Agonists and antagonists of AhR as well as new strategies for treatment using the microbiome–AhR axis are summarized. JAK, Janus tyrosine kinase; STAT, signal transducer and activator of transcription; EGFR, epidermal growth factor receptor; HIF‐1α, hypoxia inducible factor 1 subunit alpha; SLE, systemic lupus erythematosus; RA, rheumatoid arthritis; SS, Sjogren's syndrome; MS, multiple sclerosis; T1DM, type 1 diabetes mellitus; MN, membranous nephropathy; UC, ulcerative colitis; AD, atopic dermatitis. Created with www.figdraw.com.

In this review, we begin by exploring the fundamental structure and commonly associated ligands of AhR. This is followed by a comprehensive analysis of its genomic and nongenomic signaling pathways, with particular attention to the interactions between these pathways. The review primarily concentrates on elucidating the role of AhR in modulating immune cells within both innate and adaptive immunity, thereby establishing a connection between AhR‐mediated regulation of immune cells and its clinical implications across various diseases. These diseases predominantly include autoimmune disorders, inflammatory conditions, tumors, and neurodegenerative diseases. We provide a synthesis of clinical trials targeting AhR therapeutically, introduce a novel therapeutic strategy centered on the microbial–AhR axis, and highlight the significant potential for AhR development. This review aims to furnish theoretical support for the precise formulation of regulatory strategies (Figure 1).

## 2 Molecular Mechanisms of AhR Signaling

### 2.1 Structural Insights into AhR Activation

AhR functions as a transcription factor and environmental sensor, exhibiting widespread expression in barrier organs including the skin, intestines, and lungs, as well as in various immune cell types such as dendritic cells (DCs), macrophages, T cells, and B cells [6]. The human AhR gene is located on chromosome 7. As a member of the basic helix–loop–helix (bHLH) Per–Arnt–Sim (PAS) family, AhR is vital for immune system function and cellular homeostasis. Structurally, AhR can be divided into three main regions: the N‐terminal bHLH domain, the C‐terminal variable domain, and the PAS domain [7]. The bHLH domain is essential for DNA binding and facilitates AhR interaction with DNA response elements (DREs) [8]. The C‐terminal region of AhR contains a variable transcriptional activation domain, which is responsible for initiating the transcription of target genes [9]. The PAS domain consists of a concatenated PAS‐A and PAS‐B subdomains, serves as the ligand recognition region of AhR and is characterized by five β‐strands and four α‐helices [10]. Under steady conditions, AhR and the chaperone protein Hsp90, the cochaperone p23, and the interacting protein XAP2 form a complex in the cytoplasm [11]. Hsp90 interacts with AhR through a hydrophilic interaction site located on the PAS‐B domain, thereby protecting AhR from degradation, maintaining its stability in the cytoplasm, and concealing its nuclear localization sequence (NLS). This inactivation further promotes the enhancement of AhR ligand‐mediated signaling activation [12]. Additional cytoplasmic proteins, such as XAP2 and p23, which effectively prevent ubiquitination and degradation, contribute to the stabilization of AhR [13]. These components collectively form the AhR complex, thereby facilitating its functional role in biological organisms.

The structural basis for the diversity of AhR ligands is attributed to the hydrophobic pocket within the PAS‐B domain, which exhibits a notable degree of plasticity [14]. The particular amino acid residues within the PAS‐B pocket are capable of interacting with various ligands, thereby partially influencing the affinity and specificity of ligand binding. Histidine 291 in humans is crucial in configuring the ligand binding site, with the side chain of the imidazole loop exerting a direct influence on ligand binding affinity through steric effects [15].

**FIGURE 2:**
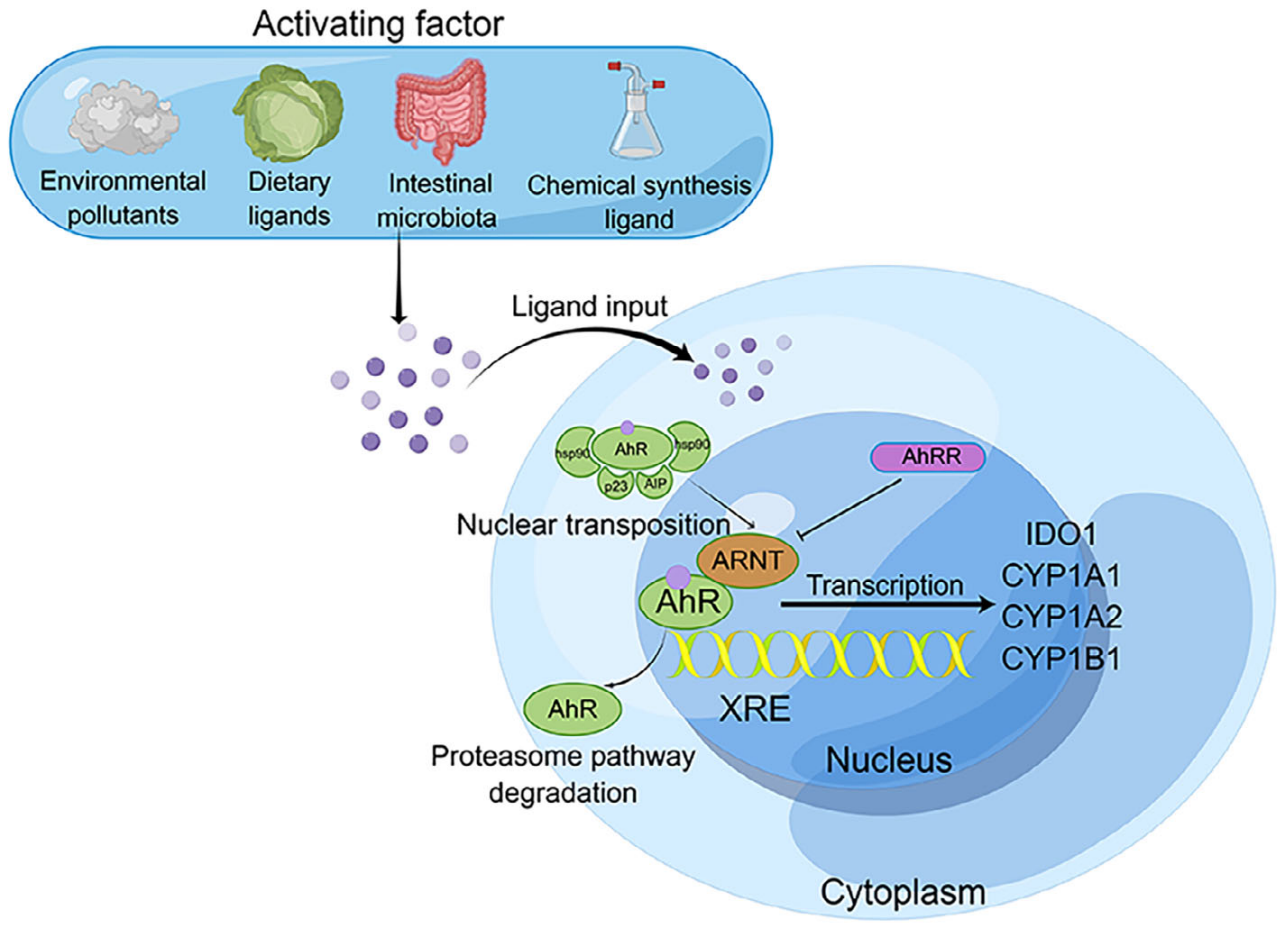
Mechanism of the aryl hydrocarbon receptor activation. AhR ligands from environmental pollutants, dietary intake, and intestinal microbiota activate intracellular AhR, and the formed AhR–ligand complexes enter the nucleus through the nuclear pore. In the nucleus, the complex dimerizes with the aryl hydrocarbon receptor nuclear transporter protein (ARNT), which in turn binds to the xenobiotic response element (XRE) on DNA and promotes the expression of downstream genes, while the AhR is degraded via the proteasome pathway. Aryl hydrocarbon receptor repressor (AhRR) negatively regulates this process. IDO1, indoleamine 2,3‐dioxygenase 1; CYP1A1, cytochrome P450 family 1 subfamily A member 1; CYP1A2, cytochrome P450 family 1 subfamily A member 2; CYP1B1, cytochrome P450 family 1 subfamily B member 1. Created with www.figdraw.com.

AhR ligands from environmental pollutants, dietary intake, and intestinal microbiota activate intracellular AhR, there is an observed leakage of NLS. Consequently, AhR dissociates from the complex and is translocated through the nuclear pore complex into the nucleus, where it dimerizes with the AhR nuclear translocator (ARNT). The resulting AhR–ARNT dimer is able to recruit many accessory transcription factors and bind to the DREs, thereby promoting the expression of downstream genes [16]. This metabolic involves enzymes like cytochrome P450 family 1 subfamily A member 1 (CYP1A1), cytochrome P450 family 1 subfamily A member 2 (CYP1A2), cytochrome P450 family 1 subfamily B member 1 (CYP1B1). Also involved AhR repressor (AHRR), which is a negative regulator (Figure 2) [17].

### 2.2 The Diverse Universe of AhR Ligands: From Toxins to Physiological Mediators

AhR is a ligand‐dependent transcription factor recognized for its role in modulating various biological processes through its interaction with multiple ligands. Structurally diverse compounds originating from environmental sources, dietary intake, the microbiome, and host metabolism are known to modulate AhR activity [18]. In mammals, a substantial array of AhR ligands has been identified, which can be categorized into exogenous and endogenous ligands based on their origin [19].

Exogenous ligands typically exhibit high affinity and are predominantly associated with toxicity, making them a primary focus of study within the field of toxicology. Polycyclic aromatic hydrocarbons (PAHs) and halogenated aromatic hydrocarbons represent the predominant exogenous ligands for AhR [11]. Among the environmental pollutants produced by the combustion of organic materials in industrial processes, 2,3,7,8‐tetrachlorodibenzo‐p‐dioxin (TCDD) is identified as one of the most potent agonists of AhR. The primary route of human exposure to TCDD is through the consumption of contaminated food sources. However long‐term exposure to TCDD is potentially carcinogenic [20, 21]. Additional exogenous ligands encompass polychlorinated biphenyls and benzo[a]pyrene, both of which tend to bioaccumulate and persistently activate AhR, thereby inducing toxicological effects.

The range of endogenous ligands is more comprehensive, primarily comprising metabolic products of the host organism itself and metabolic ligands derived from gut microbiota. Tryptophan metabolites are the main source of AhR ligands in vivo and play an important linker role in metabolism and immunity. Tryptophan is mainly metabolized through the kynurenine pathway, catalyzed by indoleamine 2,3‐dioxygenase (IDO) and tryptophan 2,3‐dioxygenase (TDO). Kynurenine and its derivatives produced by this pathway can interact with AhR within tumor microenvironment and immune tolerance [22]. 6‐Formylindolo[3,2‐b]carbazole (FICZ), produced from tryptophan under conditions of oxidative stress or UV light exposure, is also a high‐affinity agonist [23]. 2‐(1′H‐indole‐3′‐carbonyl)‐thiazole‐4‐carboxylate methyl ester (ITE), a nontoxic AhR agonist, is hypothesized to be a derivative of tryptophan generated under conditions of oxidative stress [24].

In addition to the metabolites produced by the host, metabolites of the intestinal microbiota can also mediate interactions. Indole‐3‐acetic acid (IAA), indole‐3‐aldehyde (IAld), indole‐3‐lactic acid (ILA), and short‐chain fatty acid (SCFA), all of which are gut‐derived metabolites, can activate the AhR to exert a regulatory role in the organism [25]. There are also many AhR ligands in dietary ingredients, which are diverse in structure and safer. Natural dietary compounds such as 3,3′‐diindolylmethane (DIM), quercetin, curcumin, indirubin, and resveratrol provide targets for future nutritional interventions. Microbiota and foodborne ligands serve as critical mediators connecting the environment, diet, gut microbiota, and host health, thereby playing a pivotal role in regulating intestinal immune homeostasis [26].

Furthermore, various pharmacological agents and synthetic modulators have been developed to leverage AhR pathway for therapeutic applications, while avoiding potential toxic effects. The ligand diversity of AhR partly determines the dual role of AhR in health and disease, which can not only mediate the action of environmental toxins, but also regulate the body immune homeostasis. This diversity has culminated in a promising therapeutic strategy, namely, the development of AhR modulators, which are expected to create new drugs with precise targeting and few side effects.

### 2.3 Canonical (Genomic) and Noncanonical (Nongenomic) Signaling Pathways

#### 2.3.1 Canonical (Genomic) Signaling Pathways

Since the discovery of AhR as a dioxin‐mediated receptor, its genomic signaling pathway (canonical signaling pathway) has been clearly elucidated. As previously discussed, ligand binding facilitates the release of AhR from the complex, enabling its translocation into the nucleus. Once in the nucleus, AhR undergoes dimerization and subsequently binds to the promoter or enhancer regions of target genes, thereby initiating transcription. This is the most characteristic pathway of AhR, dependent on DNA binding and transcriptional activation. Concurrently, AHRR functions as a negative regulator of AhR signaling pathway, thereby preventing an excessive response of AhR to agonists. This self‐limiting feedback mechanism is crucial for the maintenance of immune homeostasis within the body [27].

#### 2.3.2 Epidermal Growth Factor Receptor Pathway

AhR is also involved in nongenomic signaling pathways that operate independently of AhR–ARNT dimerization and DRE binding. These pathways influence gene expression patterns and associated physiological or pathophysiological functions in a manner dependent on the ligand, cell type, and microenvironment. Epidermal growth factor receptor (EGFR) is an important receptor tyrosine kinase. The activation of AhR by ligands facilitates signaling events driven by c‐Src, either by directly activating EGFR or by altering its conformation to initiate downstream pathways, such as the mitogen‐activated protein kinase (MAPK), Akt‐phosphoinositide 3‐kinase (PI3K)–mechanistic target of rapamycin, and nuclear factor kappa‐light‐chain‐enhancer of activated B cells (NF‐κB) pathways. Most of them are related to cell proliferation, migration, and invasion [27, 28].

To exploit the AhR‐mediated regulation of EGFR function, it is advisable to consider EGFR‐targeted therapies in conjunction with AhR antagonists as a strategy to counteract tumor progression in certain cancer types. Research has demonstrated that the suppression of AhR‐mediated expression of the MMP‐1 can inhibit the EGFR–PI3K/Akt signaling pathway, thereby contributing to the amelioration of colorectal cancer [29]. Inhibition of AhR signaling in drug‐resistant non‐small cell lung cancer disease models effectively prevents tumor growth and the development of resistance to EGFR tyrosine kinase inhibitor, ultimately improving prognosis [30]. Inhibition of this signaling pathway promotes the adhesion of apical junction proteins, which helps to maintain the integrity of the airway epithelial barrier [31]. The evidence presented indicates that the complex interaction between AhR and EGFR holds significant implications for therapies aimed at targeting EGFR and its associated signaling pathways.

#### 2.3.3 Janus Tyrosine Kinase–STAT Pathway

Janus tyrosine kinase (JAK)–STAT pathway is a common cascade that gradually transposes cell surface signals into the nucleus and participates in a variety of physiological and pathological processes [27]. The effect of AhR on the JAK–STAT pathway is mainly mediated by the expression of various cytokines. After activation of AhR by ligands, AhR translocate to the nucleus and form heterodimers, driving the transcription of AhR target genes and regulating the expression of different JAK/STAT‐stimulated cytokines. Including IL‐2, IL‐10, IL‐21, IL‐22, and so on. These cytokines activate JAKs via their receptors, leading to receptor phosphorylation and STAT docking site formation. Then, STAT dimerization enters the nucleus and binds to DNA, which is the most direct and major regulation mode of AhR on this pathway.

AhR agonist β‐nafflavone can inhibit the production of autocrine IL‐6, promote the activation of STAT3, and induce the differentiation of glial cells [32, 33]. The activation of STAT5 by IL‐2 within the tumor microenvironment leads to the depletion and functional impairment of CD8^+^ T cells [34]. In addition, AhR can also mediate the activation of interferon‐γ (IFN‐γ) through JAK–STAT, upregulate PD‐L1 and IDO1, and the produced Kyn and its metabolites in turn act as AhR agonists to induce regulatory T (Treg) immunosuppression [35]. This suggests that STAT protein is one of the important factors for the functional integrity of AhR. Bidirectional regulation of AhR and JAK–STAT pathways is an important mechanism by which AhR orchestrates immune responses.

#### 2.3.4 E3 Ubiquitin Ligase Activity

An additional significant aspect of nongenomic signaling pathways is the role of AhR as an E3 ubiquitin ligase, facilitating the proteasomal degradation of proteins, including steroid receptors [36]. It serves a critical role in substrate recognition by utilizing its ligand‐binding domain to identify proteins designated for degradation and facilitating the ubiquitination of these target proteins. It regulates multiple key pathways in the body through cross‐talk and mediates ubiquitination and proteasomal degradation of a variety of receptors and signaling proteins, which also determines the functional diversity of AhR.

### 2.4 Cross‐Talk With Other Signaling Pathways

#### 2.4.1 Cross Interaction With NF‐κB

The NF‐κB pathway consists of classical and nonclassical pathways and plays a role in a variety of physiological and pathological processes. After activation, the classical pathway is involved in the occurrence of inflammation, immune response, cell proliferation and differentiation, and other events, regulating the expression of various proinflammatory genes and responding more rapidly. The activation of noncanonical NF‐κB is slow but persistent and also plays an important role in the immune system and immune response [37].

AhR functioning as a transcription factor that engages with NF‐κB signaling plays a pivotal role in modulating immune responses and exhibits significant cross‐talk with the NF‐κB signaling pathway [38]. The inhibitory effect of AhR on NF‐κB signaling has been extensively studied. AhR activation inhibits NF‐κB activity and its mediated inflammatory response. The primary mechanism involves a direct physical interaction between AhR and RelA, a subunit of NF‐κB, which inhibits the transcription of genes downstream of RelA [39, 40]. In the context of infection or inflammation, endogenous AhR ligands are produced by the organism to prevent further amplification of disease effects through the regulatory effects described above [41]. AhR activation also promoted the degradation of RelA.

Conversely, the activation of NF‐κB also alters the expression and activity of AhR. Prior research has shown that the activation of drug metabolizing enzymes regulated by AhR is modulated by cytokines produced through the NF‐κB signaling pathway. Furthermore, the activity and expression of these drug‐metabolizing enzymes are inhibited following the intervention of TNF‐α and IL‐1β [42, 43].

#### 2.4.2 Cross Interaction With HIF‐1α

HIF‐1α and AhR are members of the PAS protein family. Under normoxic conditions, HIF‐1α protein is hydroxylated and degraded by proteasome. In contrast, under hypoxic conditions, these enzymes are inactive and HIF‐1α enters the nucleus and dimerizes with ARNT, which then binds to the hypoxia response element in the enhancer/promoter region of target genes [44]. This phenomenon resembles the classical pathway response of AhR. When HIF‐1α pathway is activated, it competes with the AhR pathway for ARNT, thereby partially inhibiting the AhR pathway [45, 46].

However, numerous studies have demonstrated that the primary mode of interaction is synergistic, indicating that the activities of HIF‐1α and AhR are interdependent across various tissues. The cellular concentration of ARNT is relatively abundant, thereby not constraining the binding efficiency of the dimeric protein [47]. The cross‐talk between AhR and HIF‐1α pathways exists in a variety of tissues and cells in vivo, facilitating the integration of environmental stimuli into cellular responses. For example, in epidermal keratinocytes, the activity of HIF‐1α is persistently diminished in the absence of mild hypoxic conditions within ischemic epidermal regions. Furthermore, DNA repair processes in these keratinocytes predominantly rely on the activity of AhR [48]. In glioblastoma, HIF‐1α and AhR orchestrate the metabolic reprogramming of lymphocytes, modulate tumor‐specific immune responses, and facilitate malignant tumor progression [49]. These examples suggest that the synergy of AhR with the HIF‐1α pathway is not a simple additive benefit and needs to be analyzed according to the specific context to become a viable therapeutic strategy.

#### 2.4.3 Cross Interaction With Estrogen Receptor Signaling Pathways

AhR and estrogen receptor (ER) pathways play integral roles in mammalian reproductive processes, with intricate interactions encompassing metabolic processes, transcriptional activity, protein stability, and epigenetic regulation [50]. Understanding the interaction pattern between the two pathways will help us to develop precision medicine targeting this pathway in the future.

Activation of AhR modulates the activity of hepatic enzymes CYP1A2 and CYP3A4, as well as various metabolic enzymes in extrahepatic tissues. This enzymatic activity facilitates the degradation of endogenous estrogen in vivo, ultimately resulting in decreased hormone levels [51]. Activation of AhR by heterologous ligands leads to the disruption of ER signaling at the transcriptional level. The AhR/ARNT complex binds to specific promoters, where the associated DNA sequences function as inhibitory dioxin‐response elements, exemplified by cathepsin D [52]. The antiestrogenic effect of AhR is significantly influenced by its E3 ubiquitin ligase activity. This activity facilitates the recruitment of target proteins, including ER and other steroid hormone receptors, for ubiquitination and subsequent degradation. Notably, this function operates independently of AhR dimerization with ARNT [53]. In summary, AhR is a powerful negative regulator of the ER pathway.

## 3 Physiological Functions of AhR

Initially identified as a receptor for dioxins, AhR is primarily associated with the detoxification and metabolism of environmental carcinogens [54]. Diverse physiological functions of AhR have been identified, and studies have shown that AhR plays an important role in participating in the immunoregulatory network, the developmental differentiation of embryonic tissues, and metabolic functions [55]. Incorporating the latest advancements in AhR research is essential for developing a comprehensive understanding of its functions, thereby enhancing both the theoretical knowledge and practical applications of AhR.

### 3.1 AhR Regulates Immune Cell Development and Function

**FIGURE 3:**
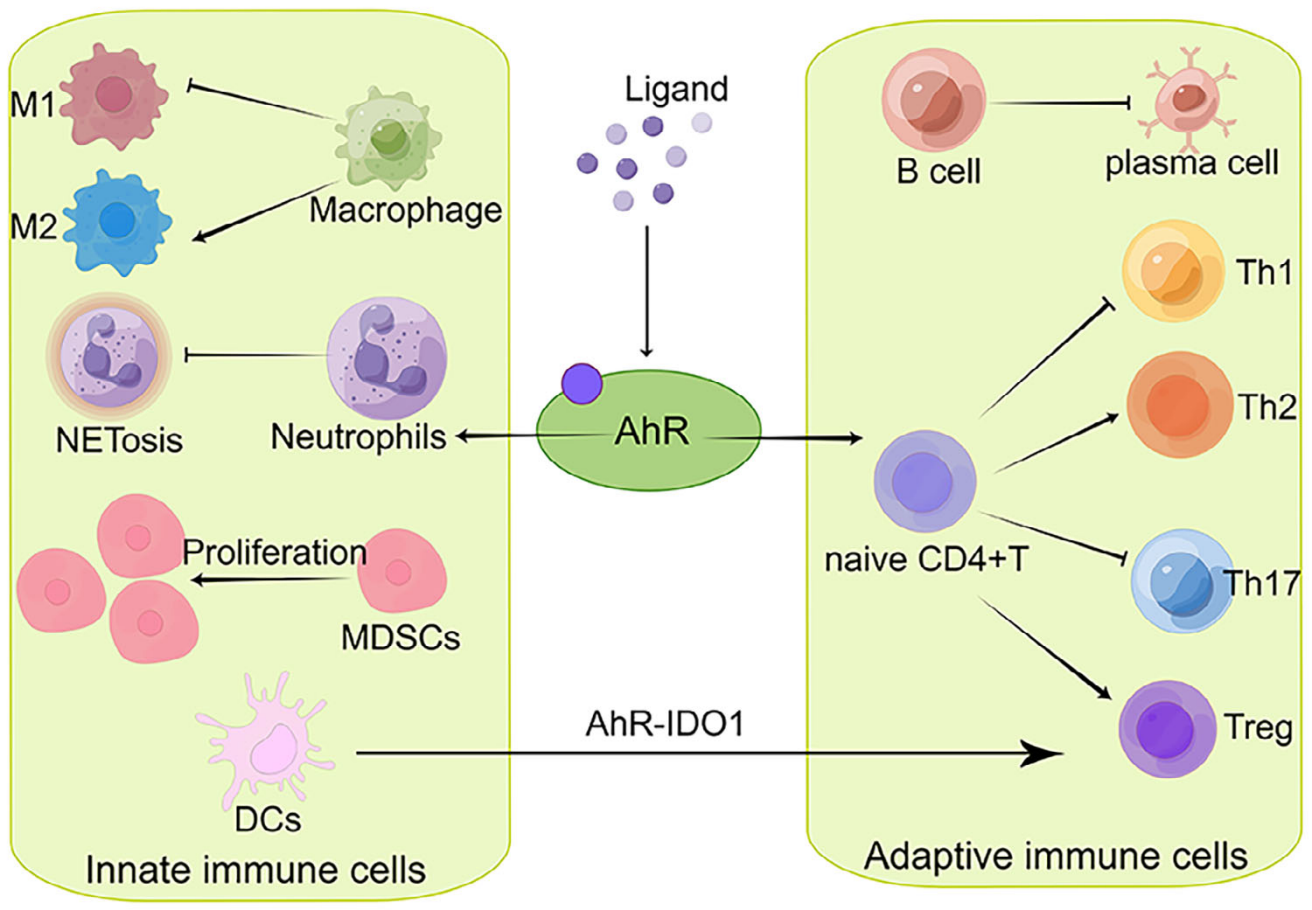
Regulation of immune cells by activated AhR. Upon activation by the ligand, AhR can regulate various immune cells of innate and adaptive immunity. AhR is able to influence CD4^+^ T cell differentiation. Activated AhR can also have an effect on B cell differentiation, reducing B cell differentiation to plasma cells and autoantibody production. Activated AhR can also have an effect on macrophage polarization, with a shift from a proinflammatory M1 type to an anti‐inflammatory M2 type. Activated AhR also induces a large accumulation of myeloid‐derived suppressor cells (MDSCs), exerting its immunosuppressive effects. It also has an effect on the developmental differentiation of dendritic cells (DCs) and neutrophils. NETosis, neutrophil extracellular traps; Th1, T helper cell 1; Th2, T helper cell 2; Th17, T helper cell 17; Treg cells, regulatory T cells. Created with www.figdraw.com.

Numerous studies have demonstrated that AhR signaling is pivotal in modulating both innate and adaptive immune responses (Figure 3). Within the realm of innate immunity, AhR is instrumental in governing the functions of natural killer (NK) cells, DCs, and macrophages, thereby influencing their ability to recognize and respond to pathogens threats [56, 57]. Regarding adaptive immunity, AhR significantly contributes to the differentiation, proliferation, and antibody production of T and B lymphocytes, particularly in the regulation of the T helper cell 17 (Th17) and Treg cells balance [58]. Furthermore, AhR activation can alter the secretion patterns of immune cytokines, thus modulating both local and systemic immune responses [7]. This also establishes the central role of AhR in maintaining immune homeostasis.

#### 3.1.1 B Cells

B cells are acknowledged as a sensitive target for the postactivation effects of AhR. The expression of AhR varies across different B cell subsets, with lower levels observed in bone marrow precursor B cells and higher levels in spleen transitional B cells and plasma cells. This expression pattern implies that AhR may be instrumental in the maturation and functional regulation of B cells. Recent studies have demonstrated that the mechanisms by which AhR influences B cells operate at multiple levels, encompassing B cell maturation, differentiation, and the regulation of immune responses [19]. AhR plays a significant role in the differentiation of B cells, influencing their fate by regulating class switch recombination and plasma cell differentiation [59]. AhR mediates the differentiation of B cells from hematopoietic stem cells into pre‐B cells, mature B cells, and plasma cells, which includes negative effects, such as preventing the differentiation of B cells into plasma cells when AhR is overactivated [60].

The activation of AhR is crucial for B cell proliferation. Research indicates that B cells lacking AhR exhibit diminished proliferative capacity following B cell receptor (BCR) stimulation in vitro and face challenges in progressing to the S phase of the cell cycle. AhR‐deficient B cells are competitively disadvantaged, impeding their ability to effectively execute antigen‐dependent proliferative responses in vivo [61].

Activation of AhR promotes the regulation of immunoglobulin heavy chain (IgH) genes through a direct transcriptional mechanism [62]. Modulation of AhR activity can alter the expression profile of IgH isotypes and the secretion of specific antibody isotypes. In animal models, activation of AhR by TCDD typically results in the suppression of antibody secretion [63]. In various subpopulations of human B cells, AhR activation exerts differential effects on Ig isotype expression at the transcriptional level. CD5^+^ B cells, also referred to as innate immune‐like B cells, are crucial for immune function, primarily through the continuous secretion of IgM [64]. CD5^+^ B cells are particularly susceptible to TCDD‐induced IgM suppression. When isolated from the CD19^+^ pool, CD5^+^ B cells exhibited a greater degree of CD40 ligand‐induced IgM suppression compared with CD5^−^ B cells following TCDD exposure. Conversely, in the human Burkitt lymphoma B cell line, IgM and IgA secretion remained unaffected, whereas IgG secretion was significantly inhibited [63]. Mouse B‐1 cells are similar to human CD5^+^ B cells. The ability of B‐1 cells present in the mesothelial lumen to produce antibodies is non‐T cell dependent, and AhR decreases the ability of B‐1 cells to secrete antibodies, instead favoring their immunosuppressive activity [65]. ITE inhibited not only the expression of IgM, but also IgG1 and IgE. ITE was also found to inhibit the expression of secreted IgM RNAs and plasma cell‐specific genes [66]. These data suggest ligand and species variability in AhR function in B cells.

At the cellular signaling level, AhR activation can interact with other signaling pathways, including the ER signaling pathway and MAPK pathway, which are essential for B cell maturation [67]. Those signaling pathways can alter the composition of B cell subpopulations, such as enhancing the function of regulatory B (Breg) cells, which play a suppressive role in immune regulation [68]. In conclusion, AhR exerts a distinct regulatory influence on the expression profile of antibody isotypes in human B cell lines, potentially playing a significant role in modulating immune responses and associated diseases.

#### 3.1.2 T Cells

Within the intricate network of the immune system, T cells assume a pivotal role. Activation of AhR affects various biological processes in T cells, such as cytokine production, T cell receptor signaling, and the homeostasis of T cell subpopulations. AhR is now recognized as a key regulator of T cell immunity.

AhR expression is most pronounced in Th17 and Treg cells, while it is either absent or minimally expressed in naïve T cells [69]. Research indicates that the activation of AhR significantly influences the development of Th17 cells, primarily through STAT signaling pathway. STAT3 interacts with AhR and coregulates T cell differentiation [70]. AhR may also regulate STAT1 activation by acting as a ligand‐dependent E3 ubiquitin ligase in the generation of Th17 cells by degrading activated STAT1 [71]. AhR facilitates the induction of Th17 cells by interacting with STAT5 and subsequently attenuating the activation of STAT5 mediated by IL‐2 in naïve T cells. Several AhR natural ligand bodies have been shown to promote STAT5 activity and inhibit Th17 differentiation [72].

In addition to signaling pathway drivers, cytokines produced during AhR activation also promote T cell differentiation. The AhR–STAT1 pathway may affect Th17 differentiation by regulating cytokines such as IL‐6 and TGF‐β [73]. IL‐23 is also essential for Th17 differentiation, and when this cytokine is absent, Th17 cells may be converted to IL‐10‐producing immunosuppressive Tr1 cells [20]. AhR regulates epigenetic modifications of Th17 cell‐related genes through interaction with RORγt, thereby affecting Th17 cell differentiation and function [7]. Overall, AhR is an important regulator of Th17 function.

Treg cells are crucial mediators of immune tolerance [74]. Research has demonstrated that AhR can augment the expression of Foxp3 mRNA [75, 76]. In addition to directly enhancing Foxp3 transcription, AhR also epigenetically regulates Treg cell differentiation. Activation of AhR by TCDD leads to demethylation of CpG islands present in the FOXP3 promoter, allowing Treg cell to differentiate preferentially to play an immunosuppressive role [77]. At the cytokine level, iTreg cell differentiation is dependent on high concentrations of TGF‐β. In addition to this, Treg cell differentiation cannot be achieved without the critical role of DCs, and AhR can affect Treg cell differentiation and function by regulating the metabolism of IDO in DCs [78].

**FIGURE 4:**
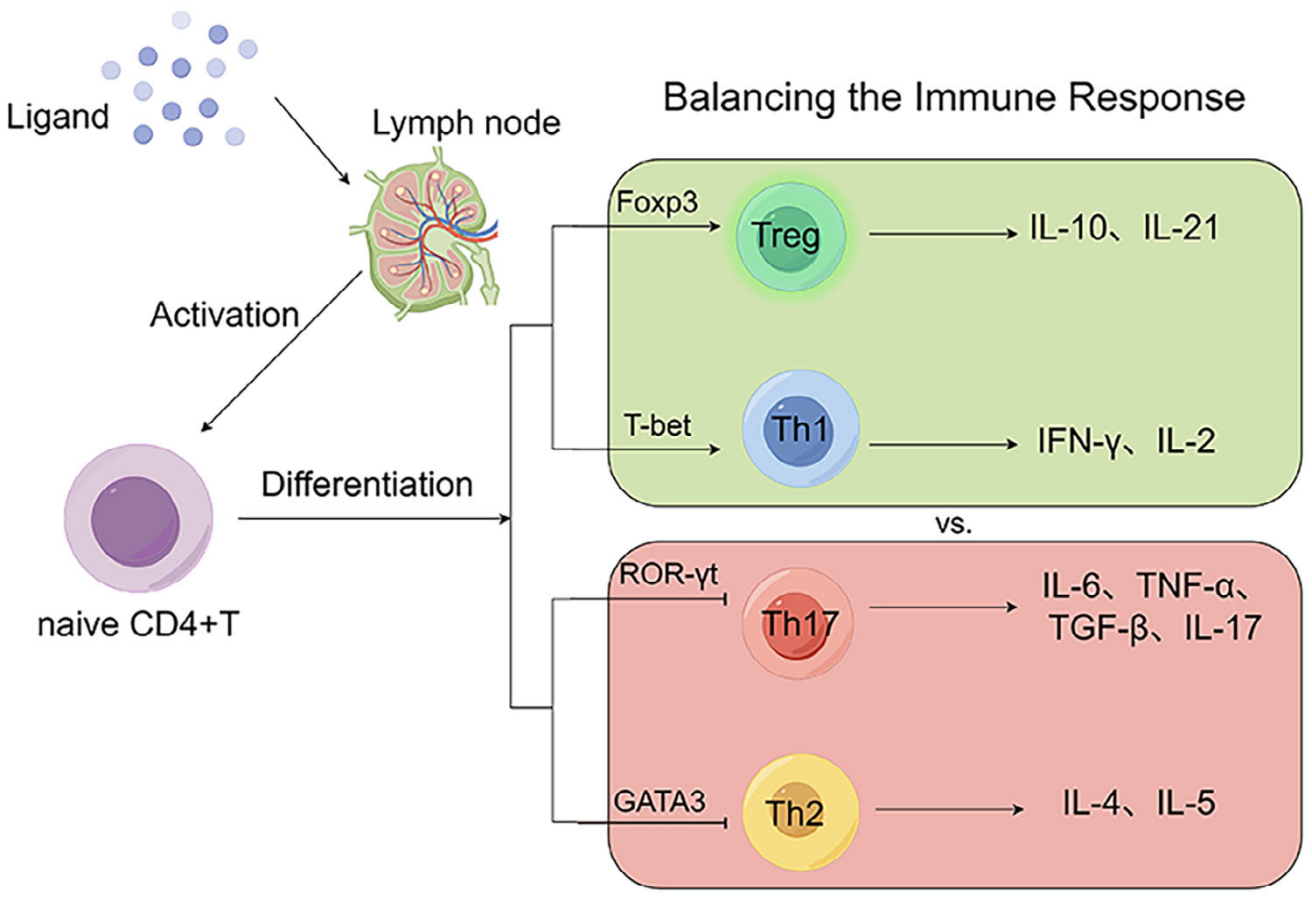
Mechanisms of the effect of activated AhR on naive CD4+ T cell differentiation. Naive CD4^+^ T cells can differentiate into a wide range of helper T cells, and activation of AhR primarily affects the balance between Treg cell and Th17, Th1, and Th2. AhR inhibits Th17 differentiation as a means of reducing inflammatory cytokine production. Meanwhile, AhR can promote differentiation to Treg cell and enhance the expression of anti‐inflammatory cytokines. AhR upregulates the expression of the transcription factor GATA3 and downregulates the expression of T‐bet, regulating the balance of Th1/2 cell production and maintaining immune homeostasis. Created with www.figdraw.com.

AhR modulates the equilibrium between Th1 and Th2 cells by influencing the differentiation of Th cells from naïve T cells [79]. Activation of AhR inhibits the differentiation of Th1 cells and the secretion of its characteristic cytokine IFN‐γ, while promoting the differentiation of Th2 cells and the production of cytokines such as IL‐4 and IL‐5. The antiallergic compound M50354 binds and activates AhR and is inhibitory to Th2 cell differentiation, making AhR signaling a promising target for the treatment of allergic diseases [80]. AhR activation has an impact on CD4^+^T cell differentiation, which greatly balances the immune response and plays an important role in inflammation and other diseases (Figure 4).

#### 3.1.3 Dendritic Cells

DCs as highly proficient antigen‐presenting cells possess the capability to effectively sample both exogenous and endogenous signals, thereby modulating adaptive immunity and maintaining a balance between the effector and regulatory components of the immune response. The activation of AhR can significantly impact the maturation, phenotype, cytokine secretion patterns, and interactions of DCs with T cells, thereby influencing the magnitude and orientation of the immune response [78].

AhR activation attenuates MHC class II expression and proinflammatory cytokine secretion in DCs [81]. DCs process antigens via MHC class II, presenting peptide–MHC complexes on their surface to sustain prolonged immune activation [82]. These complexes translocate to the DC membrane, providing a structural basis for interaction with CD4⁺ T cells. This engagement enhances TCR occupancy, promoting T cell activation and immune homeostasis maintenance [83]. Within the lymph nodes, DCs establish immune synapses with T cells, forming stable cell‐to‐cell contacts that facilitate the recognition of peptide–MHC complexes by TCR and the reception of a secondary signal from costimulatory molecules. T cell activation occurs following the reception of both the primary signal (antigen recognition) and the secondary signal (costimulatory molecules), resulting in the clonal expansion and differentiation of T cells into either effector T cells or memory T cells [84]. This process is crucial for initiating an adaptive immune response.

Activated DCs upregulate the expression of genes such as IDO1, IDO2, TGFβ1, and TGFβ3, which facilitate the establishment of an immunosuppressive environment [81]. DCs activated by AhR enhance the production of Treg cells in an IDO‐dependent manner in vitro [85]. In autoimmune diseases characterized by inflammation as the primary clinical manifestation, AhR activation can diminish the secretion of various proinflammatory cytokines by DCs, including IL‐6, TNF‐α, IL‐10, and IL‐12 [86]. This indicates that AhR is associated with the immune system through DCs to regulate immunological responses.

#### 3.1.4 Myeloid‐Derived Suppressor Cells

Myeloid‐derived suppressor cells (MDSCs) constitute a subset of myeloid cells characterized by their immunosuppressive capabilities. Under normal physiological circumstances, MDSCs undergo differentiation into mature neutrophils, macrophages, or DCs [87]. However, in pathological contexts such as tumors, inflammation, and infections, the typical differentiation process of these immature myeloid cells is impeded, resulting in the accumulation and activation of MDSCs [88].

AhR is highly expressed in human and mouse polymorphonuclear MDSC (PMN‐MDSC). A study demonstrated that the activation of AhR leads to a substantial accumulation of MDSCs, with this induction effect being partially dependent on the gut microbiome [89]. MDSCs exhibit elevated rates of mitochondrial respiration and glycolysis and display distinct microRNA expression profiles, notably with significant downregulation of miR‐150‐5p and miR‐543‐3p. These miRNAs target and enhance genes associated with anti‐inflammatory responses and MDSC regulation. Previous research has indicated that inhibiting glycolysis in vivo results in a decreased accumulation of MDSCs and a reduction in their immunosuppressive capacity, whereas AhR‐activated MDSCs increase the energy available to suppress the immune response [90, 91]. Furthermore, MDSCs influence T cell proliferation and can induce Treg cells by disrupting the Th17/Treg cell balance, thereby promoting a shift in the immune response from inflammation to tolerance [92].

#### 3.1.5 Macrophage

Macrophages can be divided into two categories according to their immune function, the proinflammatory M1 phenotype and the anti‐inflammatory M2 phenotype. M1 is involved in the Th1 immune response and secretes proinflammatory factors, while M2 is involved in the Th2 immune response [93]. AhR can regulate the balance between M1/M2 to exert anti‐inflammatory effects. Macrophages in AhR‐deficient mice produce higher levels of inflammatory cytokines such as IL‐6, IL‐12, and TNF‐α. It was also measured that their phagocytosis was reduced [94]. This suggests that alterations in the AhR gene affect macrophage polarization. An AhR agonist called Flavipin induced the expression of genes downstream of AhR in mouse CD11b macrophages and regulated the balance of pro‐ and anti‐inflammatory mediators in macrophages [95]. Decreased expression of proinflammatory factors and a shift in macrophage polarization from M1 to M2 were found after TCDD treatment of EAU mice [96]. These are associated with the downregulation of NF‐κB and STAT pathways. A novel AhR modulator, Punicalagin, a herbal monomer extract from pomegranate rind, promotes anti‐inflammatory responses in macrophages by upregulating AhR expression through the PDK1/p90RSK/AP‐1 pathway [97]. Therefore in‐depth study of the regulatory mechanisms of AhR in macrophages provides potential targets for the development of novel therapeutic strategies against inflammatory and immune‐related diseases.

#### 3.1.6 Other Immune Cells

Among the many diverse cells of the immune system, the role of AhR does not stop at the types mentioned above. NK cells, as a type of innate lymphocyte, are the core effector cells of the innate immune system. AhR activation inhibits NK cell function, which can weaken their ability to recognize tumor cells and lead to tumor immune escape [98]. Innate lymphoid cells (ILCs) are also regulated by AhR, which, in contrast to ILC1, antagonizes T‐bet and inhibits IFN‐γ secretion to reduce inflammatory injury. Balancing type 2 immune response by reducing ILC2 formation through inhibition of the Gfi1–ST2 pathway [99]. AhR can maintain ILC3 function through multiple pathways, ultimately influencing the plasticity of its subpopulations [100]. AhR activation in the gut inhibits the conversion of ILC3 to ILC1 and plays a relevant role in disease through this molecular mechanism [101, 102]. Neutrophils, which are essential in the inflammatory infiltrate, are mainly targeted by AhR to inhibit the formation of neutrophil extracellular trapping networks and prevent their over‐activation leading to tissue damage [103].

As AhR plays a central role in the immune cell network (Table 1), whether we can utilize this property of AhR to build a blueprint for human immune metabolism and achieve precise treatment of diseases in the future requires us to further unlock more functions of AhR.

**TABLE 1:** AhR key ligands and their effects on different immune cells.

Ligand	Source	Targeted cells	Mechanisms	References
TCDD (2,3,7,8‐tetrachlorodibenzo‐p‐dioxin)	Environmental pollutants	Treg cells (regulatory T cells)	Demethylation of CpG islands in the FOXP3 promoter promotes Treg cell differentiation	[77]
		Th17 (T helper cell 17)	Activation of AhR–STAT5 pathway inhibits Th17 differentiation	[72]
		B cells	Inhibition of Blimp‐1 expression, blockage of plasma cell differentiation, and inhibition of antibody secretion	[63]
M50534	Antiallergic compounds	Th2 (T helper cell 2)	Epistatic silencing of Th2 genes and impaired Th2 polarization	[80]
FICZ (6‐formylindolo[3,2‐b]carbazole)	Tryptophan photolysis product	Breg cells (regulatory B cells)	Synergizes with BCR (B cell receptor) to promote Breg cells differentiation	[104]
ITE (‐(1′H‐indole‐3′‐carbonyl)‐thiazole‐4‐carboxylate methyl ester)	Tryptophan photolysis product	B cells	Inhibits the differentiation of B cells into plasma cells, thus reducing antibody production	[66]
		DCs (dendritic cells)	Activation of AhR–IDO1 positive feedback loop promotes Treg cell differentiation	[85]
IPA (indole‐3‐propionic acid)	Metabolism of tryptophan by intestinal flora	MDSCs (myeloid‐derived suppressor cells)	Upregulation of Arg1/iNOS promotes MDSCs amplification and functional recovery	[105]
Flavipin	Fungal secondary metabolites	Macrophages	Inhibition of Arid5a/IL‐23 axis regulates inflammation and macrophage phenotype remodeling	[95]
Punicalagin	Plant extracts	Macrophages	Promotion of anti‐inflammatory responses in macrophages via the PDK1/p90RSK/AP‐1 pathway	[97]
GA (gallic acid)	Plant extracts	T cells	Balancing the Treg cell/Th17 differentiation balance	[106]
Alpinetin	Plant extracts	Treg cells	Regulation of miR‐302/DNMT‐1/CREB signaling pathway enhances Treg cell differentiation	[107]
I3A (3‐indoleacetic acid)	Lactobacillus metabolizes the product of tryptophan	ILC3 (Group 3 innate lymphoid cell)	STAT3 phosphorylation promotes IL‐22 secretion	[108]
DIM (3,3′‐diindolylmethane)	Dietary sources	Treg cells	The “glycolytic–lactate–STAT3” and TIP60 signaling pathways enhance Treg cell differentiation	[109, 110]
		Neutrophils	Inhibits the formation of neutrophil extracellular capture networks	[111]
Tapinarof	Synthetic drugs	T cells	Modulation of the JAK2–STAT3 signaling pathway hinders Tfh (T follicular helper) differentiation; expression of activated peripheral blood T cells	[112]
IAA (indole‐3‐acetic acid)	Tryptophan photolysis product	Treg cells	FOXP3 ubiquitination was attenuated by the AhR–TAZ–Tip60 pathway and Treg cell differentiation was promoted	[113]
SCFAs (short‐chain fatty acids)	Metabolites of the body	Breg cells	It is mediated by elevated levels of 5‐HIAA (5‐hydroxyindoleacetic acid)	[114]
3‐IAld (indole‐3‐carboxaldehyde)	Tryptophan photolysis product	Mast cells	Secretion of 5‐HT (5‐hydroxytryptamine) maintains autoimmune tolerance of Treg cells	[115]
AGT‐5	Derivatives of plant ingredients	ILC3	Downregulation of costimulatory molecules CD40, CD80, CD86	[116]
MSG (Moshen granule)	Traditional Chinese medicine prescriptions	Renal podocytes	Inhibits NF‐κB and Nrf2 pathways	[117]
Coal tar	Products of coal extraction	Th2	Interference with Th2 cytokines by dephosphorylation of STAT6	[118]

### 3.2 AhR Regulates Metabolism

In recent decades, beyond its role as a receptor for dioxin, AhR has been primarily recognized for its function as an exogenous sensor. This function facilitates the accelerated metabolism of various toxicants and pharmaceuticals through the cytochrome P450 enzyme system [119, 120]. A recent study suggests that clinically widely used first‐line antiepileptic drugs modulate nuclear translocation of AhR and drive transcriptional upregulation of CYP1A1, thereby affecting estrogen metabolism and calcium levels in patients [121].

Regulation of energy metabolism is one of the functions of AhR [122]. AhR has been demonstrated to modulate cellular metabolic pathways, including tryptophan metabolism, polyamine metabolism, and mitochondrial metabolic reprogramming. AhR regulates the expression of numerous genes associated with mitochondrial biogenesis and function, thereby exerting cellular effects at the transcriptional level [123, 124]. Inhibition of AhR expression adversely affects hepatic mitochondrial function, leading to a reduction in mitochondrial respiration rate and the induction of mitochondrial oxidative stress. AhR plays a crucial role in the direct regulation of mitochondrial autophagy through the mitochondrial autophagy receptor BNIP3. This process enhances and activates the mitochondrial surveillance program, thereby restoring and maintaining mitochondrial homeostasis in the liver of mice [125, 126]. In contrast, the targeted ablation of AhR in mice enhances oxidative phosphorylation in the skeletal muscle mitochondria of males, but not females. Although the mechanisms underlying the sex‐specific differences in AhR deletion are unclear, this suggests that there are different biological properties of AhR in terms of tissue and environmental specificity [127, 128, 129]. Notably, OXPHOS changes in mice with specific knockout of AhR were only maximal when mitochondria were fueled by carbohydrates [129].

In addition, AhR is also involved in the regulation of mitochondrial apoptosis. Under conditions of exogenous ligand‐induced stress, a portion of AhR translocate to the mitochondrial membrane gap, initiating its downstream signaling pathway, which is implicated in mitochondrial apoptosis within hepatocellular carcinoma cells. Furthermore, AhR is markedly overexpressed in necrotic cardiac myocytes, indicating that the AhR signaling pathway plays a crucial role in the mechanisms of apoptosis [130]. ROS‐induced mitochondrial damage exacerbates oxidative stress and activates the mitochondrial apoptotic pathway, and ROS production is largely regulated by AhR. For example, extractable organic matter in PM2.5 induces overproduction of ROS by AhR, causing endoplasmic reticulum stress resulting in developmental defects in the heart [131]. Activation of AhR by the uremic toxin indolyl sulfate promotes PGC1α degradation, inhibits mitochondrial biogenesis, and reduces ATP synthesis capacity. Disturbed energy metabolism further induces mitochondrial ROS accumulation and accelerates renal tubular epithelial cell senescence and fibrosis [132].

In summary, AhR serves as a crucial regulatory nexus linking environmental factors and metabolic processes. A comprehensive understanding of the intricate and varied regulatory mechanisms of AhR is essential for the development of therapeutic strategies aimed at targeting this receptor.

### 3.3 The Role of AhR in Organ Development

Beyond its established function as a “toxin sensor”, AhR is critically involved in embryonic development and organogenesis. AhR is involved in the regulation of the vascular microenvironment, and AhR knockout mice exhibit significant vascular developmental defects, especially in the liver [133]. Absence or hypoplasia of the portal vein is a prominent feature [134]. It should be noted that this effect was also related to the activated substance, angiogenesis was instead inhibited when TCDD activated the AhR/CYP1A1 and CYP1B1 pathways [135].

AhR also has a significant effect on cardiac physiology, although AhR is not expressed at high levels in the heart. AhR signaling is required for normal cardiac development and maturation, and it has been shown that lack of AhR in vivo results in a significant reduction in left ventricular function [136]. Adverse ventricular remodeling is a cardiac response to destructive stimuli. Some common AhR agonists, such as FICZ, ITE, and certain microbiota metabolites activate the AhR in vivo. This activation mitigates ventricular remodeling triggered by various pathological factors, thereby enhancing cardiac function [137, 138, 139, 140]. Conversely, diesel exhaust particulate, which containing high levels of PAHs, induces cardiac expression of AhR and attenuation of the HIF‐1α pathway, resulting in left ventricular dilatation and dysfunction [141]. This finding is not contradictory and aptly demonstrates the highly context‐dependent nature of the AhR in developmental processes.

AhR is expressed in a variety of neuronal cell types, including neurons, astrocytes, or microglia [142]. The disruption of AhR expression in cerebellar granule neuron precursors adversely affects neurogenesis by inhibiting the proliferation and differentiation of these precursors [143]. In the neonatal period, AhR deficiency delays the maturation of hippocampal neuronal synapses, leading to impaired hippocampus‐dependent memory and learning functions [144]. In addition to the central nervous system (CNS), myelin structures can be damaged [145].

AhR is integral to the functioning of the reproductive system, with a pronounced impact on male reproductive physiology. It influences spermatogenesis, sperm quality, and hormone regulation. Additionally, AhR may confer protective effects, although the nature of these effects is contingent upon environmental conditions. In the absence of exogenous ligands, AhR accelerates sperm G1 and S phase progression [146]. During the mitotic phase, AhR assists in the accurate segregation and movement of chromosomes, enhances the expression of nuclear membrane proteins, actin and myosin filaments, and completes the division process and the production of daughter cells [147]. During the meiotic phase, AhR is an important factor in ensuring the accurate transmission of genetic information to the next generation [148]. These highlight its importance as a regulator of male reproductive health. Chronic exposure to TCDD triggers a series of events that disrupt multiple steps in spermatogenesis and development [149].

AhR also regulates the expression of barrier‐associated proteins, which play a role in maintaining the integrity and function of barrier tissues such as the skin, gut, and lung. In lung endothelial cells, AhR facilitates pulmonary protection through apelin signaling. The absence of AhR compromises barrier integrity, thereby heightening the host susceptibility to bacterial infections [150]. These findings align with observations in the gastrointestinal tract, where an abundant presence of microbial and diet‐derived AhR ligands. This abundance may offer essential signals that contribute to the vascular AhR functions supporting barrier integrity [151, 152].

Understanding the physiological function of AhR is important for the development of interventions for related diseases, and research in this area is still ongoing. We hope to contribute to the subsequent deeper and more comprehensive development of meaningful therapeutic targets with these findings.

## 4 The Role of AhR in Various Diseases

Based on the in‐depth understanding of AhR function, a large number of studies have revealed the role of AhR in various diseases in recent years. The role of AhR in diseases presents complex character, and it promotes us to explore the underlying mechanism. This section will systematically summarize the mechanism of AhR in several common autoimmune diseases, inflammatory diseases, tumors, and neurodegenerative diseases, and help us to expand the potential of targeting AhR.

### 4.1 Autoimmune Diseases

**FIGURE 5:**
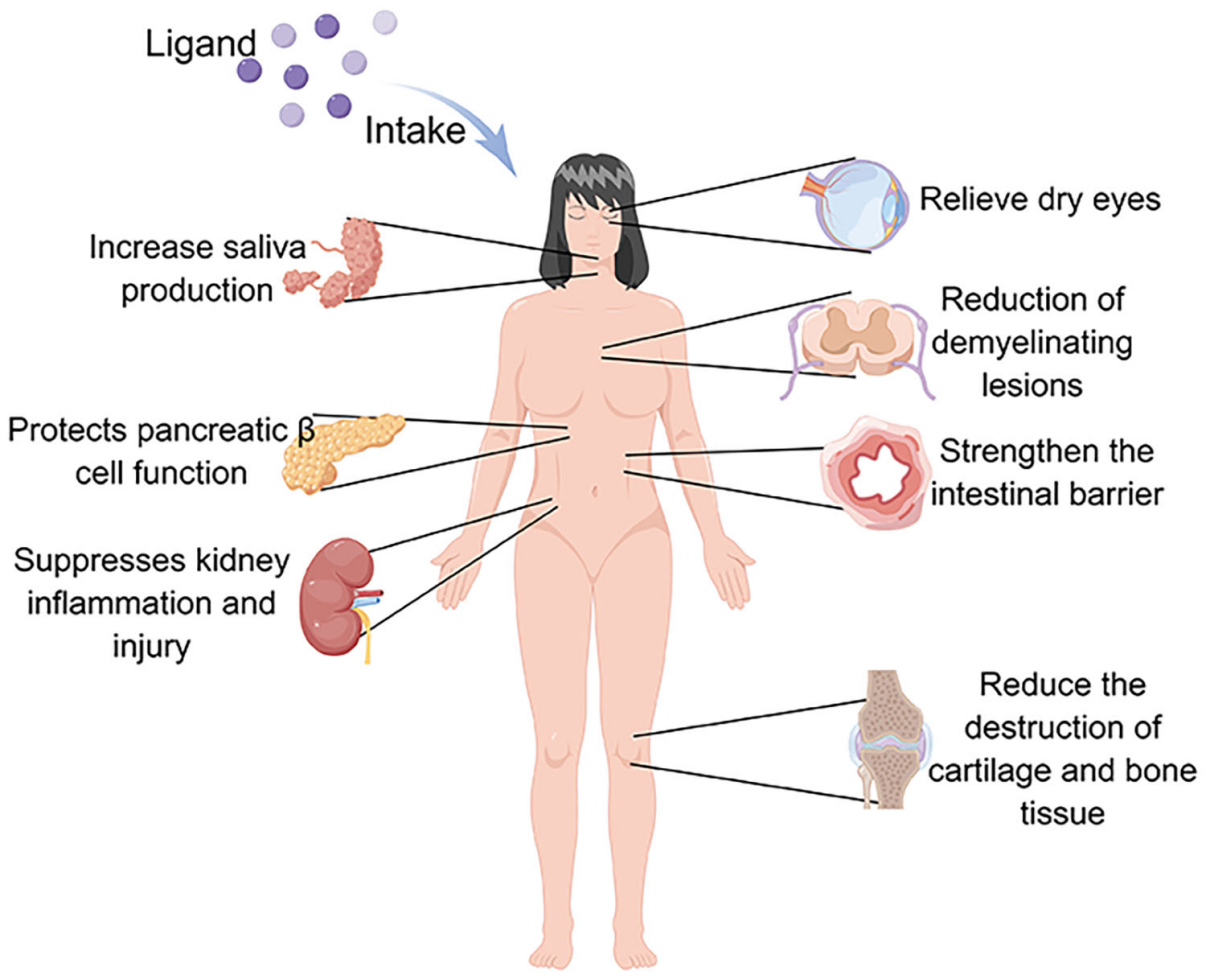
Ameliorative effects of activated AhR on several common autoimmune diseases. Activated AhR acts on immune cells through a variety of mechanisms and can mitigate the severity of the disease to some extent. Created with www.figdraw.com.

Aberrant AhR signaling has been implicated in various autoimmune diseases. In certain autoimmune diseases or their models, the administration of AhR ligands or pharmacological agents has been shown to effectively ameliorate pathological outcomes, thereby highlighting the therapeutic potential of targeting AhR in autoimmune conditions (Figure 5) [12]. A comprehensive understanding of the mechanisms underlying AhR signaling, as well as the functions of various AhR ligands or drugs, may pave the way for the development of novel treatment strategies for autoimmune diseases.

#### 4.1.1 Systemic Lupus Erythematosus

SLE is a prevalent chronic autoimmune disorder characterized by multisystem involvement. The pathogenesis of SLE is marked by aberrant immune system activation, which leads to the production of numerous autoantibodies, the formation of immune complex deposits, complement activation, and the induction of inflammation, ultimately resulting in irreversible organ damage [153].

**FIGURE 6:**
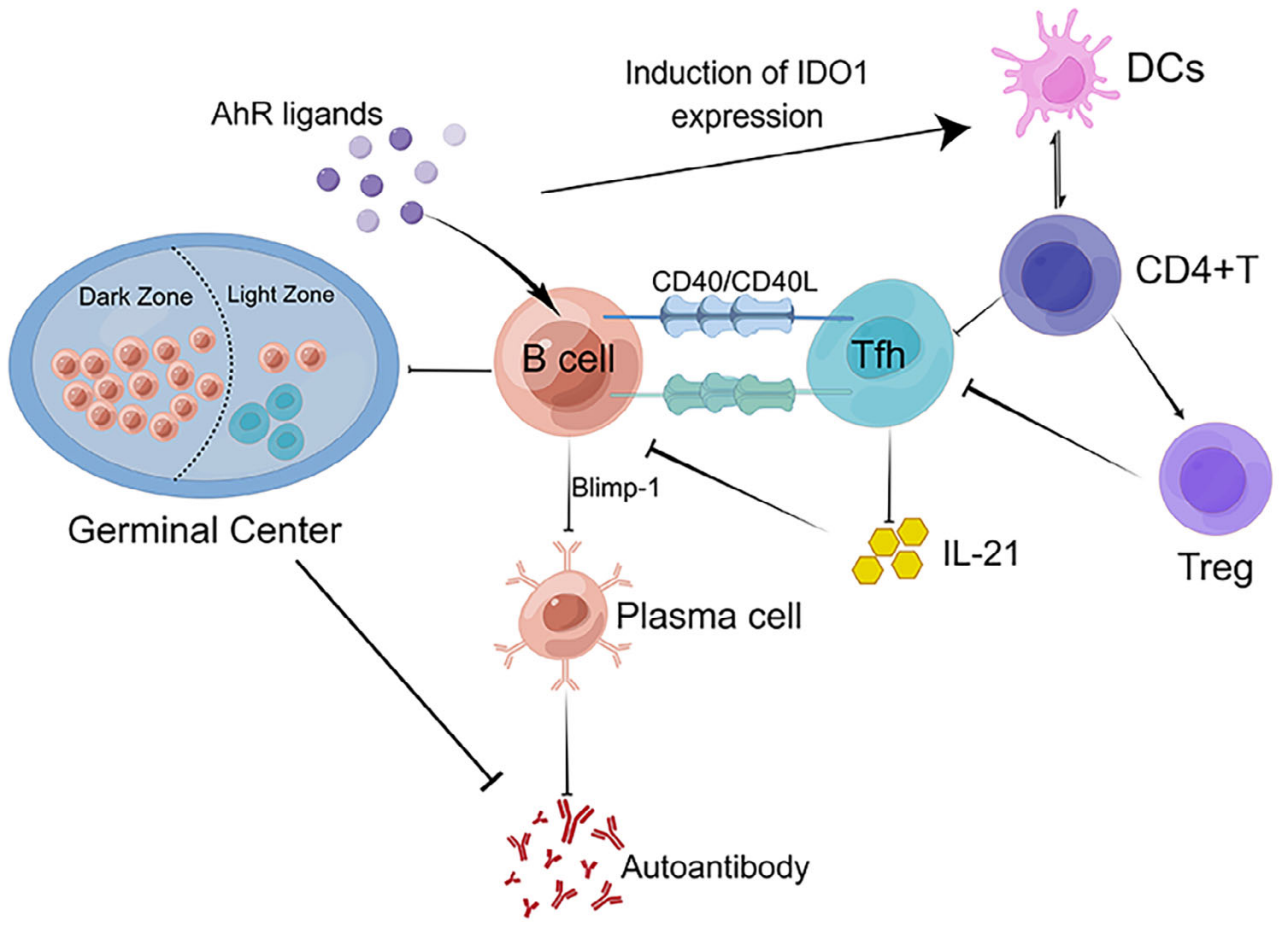
Diagram of the mechanistic role of AhR signaling in the immune setting of systemic lupus erythematosus. AhR ligands are capable of interacting with a wide range of immune cells. AhR acts with B cells and T follicular helper (Tfh) cells in the germinal center to reduce Tfh cell activation and IL‐21 production through a bridging effect of costimulatory signals, as well as inhibiting plasma cell differentiation and reducing autoantibody formation by affecting Blimp‐1 expression. On the other hand, AhR enhanced the expression of IDO on the surface of dendritic cells, and DCs and CD4^+^ T cells acted to increase Treg cell differentiation. Treg cells in turn enhance the suppression of Tfh, and the multiple effects reduce the immune response in the germinal centers to achieve remission of SLE. Created with www.figdraw.com.

The therapeutic mechanism of AhR in SLE encompasses multiple dimensions. The germinal center (GC) response in SLE is both active and persistent, constituting a principal factor contributing to the production of a substantial quantity of autoantibodies. Activation of AhR can modulate the GC response by inhibiting the CD40/CD40L costimulatory signaling pathway and diminishing the reciprocal interactions between B cells and T follicular helper (Tfh) cells. Consequently, this leads to a reduction in the differentiation of B cells into long‐lived plasma cells and attenuates the primary effector functions of Tfh cells [154, 155]. Simultaneously, T follicular regulatory cells modulate GC response and reestablish immune equilibrium. Targeting AhR in the GC response has emerged as a novel therapeutic strategy for SLE (Figure 6).

Notably, SLE is an autoimmune disease driven by pathologic T cell–B cell interactions [156]. In addition to T cells, recent research has identified several B cell subpopulations implicated in the pathogenesis of SLE, including age‐associated B cells, and initiating B cells characterized by autoreactive BCR expression, such as marginal zone B cells, B‐1 cells, and Breg cells [157, 158]. The dysregulation of these B cell subsets in SLE may be associated with the activation status of AhR. AhR in B cells correlate with SLE disease activity, and one study predicts that it could serve as a potential marker of lupus kidney injury [158]. Activation of AhR has been shown to achieve a low lupus disease activity state, thereby mitigating the risk of multiple adverse outcomes [159]. In myeloid cells, the deficiency of AhR has been demonstrated to facilitate the development of SLE [160].

Gut microbiota dysbiosis is emerging as a potential environmental component driving SLE [161]. ILC3s, as the most numerous population of ILCs in the intestine, located in the intestinal mucosa, they are key sentinels of intestinal homeostasis [162]. AhR is a transcription factor essential for the development and function of ILC3, and preclinical data have demonstrated that activation of AhR is involved in the regulation of IL‐22‐derived antimicrobial peptide (AMP) production in intestinal ILC3 [163]. Although studies on AhR modulation of intestinal ILC3 to ameliorate lupus pathology are currently underdeveloped, it is expected to serve as a therapeutic intervention therapy for SLE.

#### 4.1.2 Rheumatoid Arthritis

RA is a chronic, systemic inflammatory disease that ultimately results in the breakdown of joint cartilage [164]. The etiology and pathogenesis of RA are highly intricate, involving various aspects of immune dysfunction observable in multiple immune cell types, as well as the dysregulated secretion of proinflammatory and anti‐inflammatory factors [165].

Recent research has demonstrated that the intestinal tryptophan metabolite IAA mitigates Foxp3 ubiquitination through the AhR–TAZ–Tip60 signaling pathway. This process facilitates the differentiation and activation of Treg cells, thereby attenuating inflammation in a rat model of collagen‐induced arthritis [113]. A separate study demonstrated that patients with RA and mice with arthritis exhibited decreased levels of microbially derived SCFAs in comparison with healthy controls. Supplementation with butyrate, a specific SCFA, has been shown to mitigate the severity of arthritis in a murine model. The mechanism of action involves the activation of AhR, which is mediated through the elevation of 5‐hydroxyindoleacetic acid, a metabolite derived from serotonin.

AhR transactivation in Breg cells induces IL‐10 secretion and thus suppresses Th1 and Th17 inflammatory responses in RA model mice [114]. In addition to its effects on B and T cells, activation of AhR has been shown to attenuate the polarization of M1‐type macrophages via the AhR–Mir‐142A–IRF1/HIF‐1α signaling pathway, while simultaneously promoting the polarization of M2‐type macrophages. AhR activation may attenuate the inflammatory response in RA by modulating macrophage polarization [166]. The therapeutic mechanisms of AhR in RA encompass the regulation of immune cell polarization, modulation of the intestinal microbiota and immune cell function, and the regulation of immune responses through various protein modification pathways. Collectively, these mechanisms may contribute to the reduction of inflammatory responses and joint damage in RA.

#### 4.1.3 Sjögren's Syndrome

Primary Sjögren's syndrome (pSS) is a progressive autoimmune disorder characterized by dysfunction and damage of various organs and tissues, especially affecting the lacrimal and salivary glands, leading to pronounced symptoms of dry eye and dry mouth [167]. Previous studies have reported impaired function of MDSCs in individuals with pSS and in experimental Sjögren's syndrome (ESS) mouse models [168]. It has been shown that AhR expression in PMN‐MDSCs is attenuated during the disease process in SS and is also accompanied by reduced ROS production and diminished regulatory function. Activation of AhR results in the extensive mobilization of MDSCs exhibiting immunosuppressive properties, which play a crucial role in the regulation of proliferation, differentiation, and functional regulation of PMN‐MDSCs. This immunosuppressive effect has been validated in a murine model of Sjögren's syndrome, highlighting the therapeutic potential of AhR targeting in pSS [91].

In addition to MDSCs, one of our studies showed that AhR synergizes with BCR/TCR to promote the differentiation of Breg cells, another type of cells with immunosuppressive properties, has higher IL‐10 expression, suppresses humoral and cellular immune responses, and alleviates pathological symptoms in a mouse model of desiccation syndrome [104]. This evidence suggests that AhR signaling may serve as a novel drug target for the treatment of pSS patients.

#### 4.1.4 Multiple Sclerosis

MS is among the most prevalent autoimmune disorders affecting the nervous system, with its etiology not yet fully elucidated. Current understanding suggests associations with gut microbiota and external environmental factors [169]. It is hypothesized that MS may result from an autoimmune assault on the myelin sheath. This disease primarily impacts the CNS, comprising the brain and spinal cord, leading to neurological impairments [170].

Previous research has identified AhR as a promising therapeutic target for MS, as it ameliorates symptoms of experimental autoimmune encephalomyelitis (EAE) through the introduction of both endogenous and exogenous ligands that interact with AhR. In the right microenvironment, AhR ligand‐induced Breg cells are an important part of the T‐cell inhibitory machinery during EAE therapy [171]. Exogenous ligands, such as indole, can ameliorate EAE through a similar mechanism [172, 173]. Furthermore, dietary tryptophan is converted into a range of AhR ligands by the intestinal microbiota, including indole, indole‐3‐sulfate, indole‐3‐propionic acid (IPA), and IAId. These ligands can activate AhR signaling in astrocytes via IFN‐I signal transduction, modulate astrocyte activity, inhibit CNS inflammation, and consequently reduce the EAE disease score [174]. In addition to this, 3‐IAld has been found to induce a regulatory network in MS that causes mast cells to secrete 5‐HT to maintain self‐immune tolerance of Treg cell, reducing inflammation [175]. These studies have laid the groundwork for AhR as a preventive and therapeutic strategy for MS.

Notably, the type of T cell differentiation may depend on the nature of the AhR ligand. Activation of AhR by FICZ interferes with Treg cell development, promotes Th17 cell differentiation, and increases the severity of EAE in mice [58]. As mentioned above, the exact mechanism of this differential effect is unclear, and coupled with the high degree of T‐cell plasticity, there is still a long way to go before AhR can be used as a targeting strategy for the treatment of MS.

#### 4.1.5 Type 1 Diabetes Mellitus

T1DM is classified as an immune‐mediated disorder, characterized by the infiltration of islet‐specific autoreactive CD8^+^ T cells, macrophages, and B cells, alongside elevated levels of autoantibodies [176]. Currently, the primary therapeutic approach involves the monitoring and regulation of blood glucose levels. There is an urgent need for innovative strategies that address the autoimmune aspects of T1DM, aiming to restore immune tolerance or preserve beta cell function, thereby enhancing patient outcomes and quality of life. AhR plays diverse roles in various cells and tissues implicated in the pathophysiology of T1DM. Different ligands exert distinct effects by activating AhR in different cellular contexts, adding complexity to the understanding of T1DM pathophysiology.

Recent research has demonstrated a correlation between diet, intestinal microbiota, and the onset of T1DM [177]. The administration of *L. johnsonii* isolated from BioBreeding diabetes‐resistant rats has been shown to delay or inhibit the onset of T1DM. The underlying mechanism involves extracellular vesicles (nanovesicles) secreted by *L. johnsonii*, which mediate AhR activation. This process protects pancreatic beta cells from apoptosis and enhances insulin secretion under high glucose conditions. Additionally, it induces a shift in macrophage polarization from the proinflammatory M1 phenotype to the anti‐inflammatory M2 phenotype, thereby ameliorating T1DM symptoms in vivo [178]. Furthermore, other AhR agonists indirectly modulate immune cells, influencing T1DM onset. For instance, TCDD has been shown to prevent diabetes onset in nonobese diabetic (NOD) mice by increasing the frequency of Treg cells in the pancreatic lymph nodes [179].

The novel fluorescent AhR ligand AGT‐5 (FluoAHRL family) enhances immune tolerance in the small intestinal lamina propria by expanding Treg cell and tolerogenic DC populations while downregulating costimulatory molecules CD40, CD80, and CD86. Concurrently, it reduces pancreatic infiltration of pathogenic Th1/Th17 cells. The elevated expression levels of AhR‐regulated CYP1A1 in CD4^+^, CD8^+^, and Treg cells corroborate the AhR‐mediated effects of AGT‐5 in these cellular populations. AGT‐5 fosters a broadly immunosuppressive environment in both the pancreas and the lamina propria of the small intestine at early stages of the disease, thereby mitigating the severity of T1DM in murine models [116].

Not all AhR agonists are effective in mitigating islet inflammation associated with T1DM. The immunomodulatory phytochemical indole‐3‐carbinol (I3C), present in cruciferous vegetables, is metabolized into an AhR ligand during digestion. Research indicates that low‐level AhR activation by I3C, localized in the intestine, leads to an increase in Th17 cells and exacerbates T1DM in NOD mice. This finding contrasts with previous results where systemic activation of AhR by TCDD reduced Th17 cell populations, suggesting that both the site and degree of AhR activation are critical variables influencing these differential effects [180]. In addition to its direct or indirect effects on the effector T cell subpopulation, AhR also influences antigen presentation by DCs and macrophages. This modulation results in decreased expression and secretion of inflammatory mediators, thereby inducing a state of tolerance. AhR signal transduction plays a crucial role in regulating immune cell development and function. It achieves this by directly binding to downstream target genes or by forming complexes with other transcription factors, thereby controlling the expression of key genes essential for autoimmune responses during the progression of T1DM [181].

#### 4.1.6 Membranous Nephropathy

Membranous nephropathy (MN) is a glomerular disorder distinguished by the widespread deposition of immune complexes within the subepithelial region of the glomerular basement membrane, which is associated with thickening of the basement membrane. It represents one of the primary etiologies of nephrotic syndrome in adults [182]. The principal pathogenic mechanism underlying MN is an autoimmune response, wherein autoantigens such as the phospholipase A2 receptor and protein 7A, containing the platelet reactive protein type I domain, can elicit an IGG4‐mediated humoral immune response [183]. Moreover, genetic predisposition and environmental pollution contribute to the pathogenesis of MN. Current clinical treatment strategies commonly include supportive therapy with diuretics, renin–angiotensin system inhibitors, immunosuppressive therapy, and anti‐CD20 monoclonal antibody therapy [182]. Nonetheless, the use of immunosuppressants is associated with significant side effects and the development of drug resistance, highlighting the need for the identification of safe and effective therapeutic targets.

Moshen granule (MSG), a Chinese patent medicine, has demonstrated renal protective effects. In one study, MSG was shown to mitigate podocyte damage by inhibiting the NF‐κB and Nrf2 pathways through AhR signaling, thereby improving proteinuria and enhancing renal function in patients with idiopathic MN [117]. Traditional Chinese medicines, including the Jianpiyishen prescription, Rhubarb Fuzi Decoction, and Bupiyishen prescription, have demonstrated efficacy in ameliorating chronic kidney disease by inhibiting AhR signaling pathways [184, 185]. Similarly, *Lactobacillus species*, which influence AhR signaling through tryptophan metabolites, have been shown to exert beneficial effects on MN [186]. These findings offer novel insights into potential therapeutic strategies for the treatment of MN.

### 4.2 Inflammatory Diseases

#### 4.2.1 Atopic Dermatitis

Atopic dermatitis (AD) is an inflammatory skin disease characterized by chronic relapsing dermatitis with severe itching. AD immune dysregulation is primarily characterized by a Th2‐type inflammatory response and increased production of inflammatory cytokines such as IL‐4 and IL‐13 [187]. AhR is highly expressed in the acute lesions of AD patients, and AhR‐targeted therapies have been developed [188]. The main core mechanisms of AhR‐targeted therapy for AD are inflammation suppression and immune homeostasis regulation. Coal tar, which consists of several PAHs, acts as an AhR ligand and interferes with Th2 cytokine signaling through dephosphorylation of STAT6, improving epidermal differentiation and barrier function [118, 189]. AhR–Ovol1–Id1 signaling axis reduces neutrophil infiltration and rescues associated barrier disruption [190].

The current AhR‐targeted drug for AD, tapinarof, has been approved by both China and the United States. As a nonhormonal drug, tapinarof accurately inhibits type 2 inflammation, promotes skin barrier repair, fights oxidative stress, and regulates skin microecological balance through multidimensional interventions in the pathological process of AD. Its safety and efficiency provide a sustainable management program for AD patients, marking a major breakthrough in the field of AD treatment [191, 192, 193].

#### 4.2.2 Ulcerative Colitis

Ulcerative colitis (UC) classified as a subset of IBD, recognized as an autoimmune disorder affecting the intestinal mucosa, though its precise etiology remains unidentified [194, 195]. Current understanding suggests that genetic predispositions, immunological factors, and environmental influences contribute to the pathogenesis of IBD.

A critical factor in the development of UC is the disruption of the equilibrium between effector T cells and Treg cells. In pathological conditions, Th17 cells produce elevated levels of IL‐17, which not only stimulates the Th1 immune response but also exacerbates inflammation by inducing the secretion of additional proinflammatory cytokines, such as IL‐12 and IL‐23. These cytokines have been implicated in the initiation and progression of IBD [196]. Consequently, AhR activation has emerged as a promising therapeutic strategy to correct immune imbalance in UC by promoting Treg cell differentiation. Multiple AhR ligands showed protective effects in experimental colitis models. The dietary component l‐tryptophan affects the number and local immune homeostasis of colonic Foxp3+ Treg cells and greatly reduces the risk of developing colitis [197]. DIM, a dietary AhR ligand, promotes a shift in the balance among Th2, Th17, and Treg cells toward Treg cells, thereby ameliorating colitis through the enhancement of Treg cell differentiation via theglycolytic–lactate–STAT3 and TIP60 signaling pathways [109, 110]. Additionally, ITE has been shown to alleviate autoimmune inflammation in experimental colitis models by inducing Treg cell expression in the spleen, mesenteric lymph nodes, and colon lamina propria lymphocytes, as well as by reducing proinflammatory cytokine levels and macrophage frequency in colitis‐afflicted mice [198]. Alpinetin, a flavonoid compound derived from the seeds of Alpinia katsumadai Hayata, functions as an AhR ligand. It activates AhR to modulate the miR‐302/DNMT‐1/CREB signaling pathway, thereby enhancing the anticolitis effects through the differentiation of Treg cells [107]. FICZ has been observed to reduce the proportion of activated CD4^+^ and CD8^+^ T cell subsets, downregulate epithelial‐derived IL‐7 expression in murine models of DSS‐induced colitis, and mitigate gastrointestinal inflammation [199]. Additionally, compounds such as TCDD, 5‐aminosalicylic acid, and Norisoboldine have been demonstrated to promote Treg cell differentiation and ameliorate UC [200, 201, 202].

Pouchitis is the most common complication after ileal storage and anal anastomosis in UC [203]. Recent findings that tryptophan metabolites improve the intestinal mucosal barrier, modulate intestinal epithelial junctions, and promote goblet cell differentiation via AhR–IL‐22 pathway in mouse dextran sulfate sodium‐induced pouchitis provide a theoretical basis for the clinical application of AhR in relevant intestinal diseases [204]. In summary, the activation of AhR by appropriate ligands to regulate immune cell homeostasis represents a promising therapeutic strategy for the treatment of UC.

### 4.3 Tumor Microenvironment

AhR is a key hub in the tumor microenvironment, acting as a cytoplasmic transcription factor that broadly suppresses immune cell function. The role of AhR in the tumor microenvironment is intricate, with increasing evidence indicating that its involvement in tumor progression or suppression is contingent upon the specific tumor type and developmental stage.

In the tumor microenvironment, the immunosuppressive effects of AhR activation occur mainly in infiltrating immune cells. The current study found significant effects in innate and adaptive immunity. Prior research has demonstrated that tumor cells enhance the expression of IDO1, an enzyme responsible for catalyzing the conversion of tryptophan to Kyn. Additionally, AhR has been shown to influence the function of DCs by modifying antigen presentation and inducing the expression of both IDO1 and IDO2, thereby facilitating the production of Kyn [78, 205]. Kyn is also taken up by megakaryocyte–erythroid progenitor cells (MEP) in the bone marrow. An imbalance in MEP differentiation, favoring megakaryocyte lineage over erythroid lineage, is implicated as a contributing factor to the development of anemia and thrombocytosis in patients with intermediate‐ and advanced‐stage malignancies [206]. These findings emphasize the potential of the IDO/TDO–Kyn–AhR axis as a target for cancer immunotherapy [207]. In addition to the effects of the endogenous metabolite Kyn, clinical samples showed that patients with diffuse large B‐cell lymphoma with high AhR expression were able to upregulate the transcription factor RUNX1 in DCs, which inhibited CD8⁺ T‐cell activation and proliferation, with a significant attenuation of antitumor T‐cell responses.

Tumor‐associated macrophages are an important component of the tumor microenvironment [208]. M2‐type macrophages are widely recognized as cells with a protumorigenic phenotype and have been experimentally demonstrated to be a contributing factor to tumor invasion and poor prognosis in diseases such as hepatocellular carcinoma, pancreatic cancer, and colon cancer [209, 210, 211]. Enhanced AhR activity binds directly to the promoter of Pdl1, leading to high expression of the immune checkpoint PD‐L1 in macrophages and suppression of T cells antitumor immunity, and this formation of an immunosuppressive microenvironment correspondingly results in an increasingly poorer prognosis [212].

In autoimmune diseases we observed that AhR mainly induces Treg cell differentiation. Alterations in Treg cell number and function may be one of the reasons for the tumor‐promoting properties of AhR. For example, in human glioma sections, Kyn‐activated AhR induced Treg cells to suppress tumor‐specific CD8^+^ T cells, promoting tumor cell survival and motility [213].

In addition to T cells and macrophages, recent studies reveal the complex regulation of B cells by AhR in the tumor microenvironment. B cells usually interact with T cells aggregated in tertiary lymphoid structures (TLS). A positive feedback loop is formed in the TLS through the IL‐21/IL‐21R axis to induce differentiation of DUSP4^+^ atypical memory (AtM) B cells. Functional studies revealed that AtM B cells acquired an immunomodulatory function to suppress T cells, ultimately leading to the formation of an immunosuppressive tumor microenvironment [214].

Taken together, AhR acts like a molecular switch in response to specific signals in the TME, and this remodeling of immune cell function creates a central role for AhR in tumor immunity. We believe it is a highly promising target for cancer immunotherapy. Development of AhR antagonists to block the AhR signaling pathway or target key enzymes of the kynurenine pathway such as IDO1/TDO [215], reducing the production of endogenous AhR ligands, promises to be an effective adjuvant therapy. Multiple inhibitors targeting AhR are undergoing clinical trials; they have great potential to overcome immune resistance and modulate the tumor microenvironment (Table 2).

**TABLE 2:** AhR inhibitors in tumor diseases.

Drugs	R&D stage	Core mechanisms	Major indications	References
BAY2416964	Phase I clinical trial (NCT04069026)	Inhibition of AhR activation and nuclear translocation	Breast cancer	[216]
StemRegenin1 (SR1)	Phase II clinical trial, now terminated	Antagonizes the AhR signaling pathway	Stem cell transplantation for hematologic malignancies	[217]
KYN‐175 (IK‐175)	Phase I clinical trial (NCT04200963)	Inhibition of AhR activation and nuclear translocation	Bladder cancer, uroepithelial cancer	[218]
BAY‐218	Phase I clinical trial	Inhibition of AhR activation and nuclear translocation	Colorectal cancer	[219]
CB7993113	Phase I clinical trial	Directly binds and blocks nuclear translocation of AhR	Solid tumor	[220]
GNF351	Preclinical stage	Inhibition of the transcriptional activity of AhR	Various solid tumors	[221]
3′,4′‐DMF	Preclinical stage	Blocking AhR nuclear translocation	Breast cancer	[222]
Ezutromid	Phase II clinical trial, now terminated (NCT02858362)	Antagonizes AhR and upregulates utrophin expression	Duchenne muscular dystrophy (DMD)	

### 4.4 Neurodegenerative Diseases

AhR expression in the nervous system is characterized by cell‐specific and dynamic changes at different developmental stages [223]. In nerve cells, AhR is expressed in the early stage of development. The expression of AhR mRNA has been detected in immature mouse cerebellar granulosa cells and neural progenitor cells in the mouse hippocampus [144, 224]. In addition to neurons, AhR protein is also expressed in various glial cells, regulating the survival of neurons [225, 226]. The expression of AhR in most neurons decreases after birth, but reactivation occurs under disease conditions or induced by environmental toxins. There are various AhR ligands that can cross the blood–brain barrier and target the regulation of physiological and pathological processes. Environmental toxin ligands have high penetrability and strong neurotoxicity, which can cause neuroinflammation and degeneration [227]. Endogenous ligands such as microbiota metabolites and dietary molecules exert protective effects on the nervous system by regulating neuroinflammation and enhancing antioxidant defense functions [228, 229].

As a hub of environment–cell interaction, AhR can regulate neurological diseases through multiple mechanisms. Abnormal activation of AhR in glial cells promotes the transformation of microglia into proinflammatory phenotypes, releasing TNF‐α and NO to directly damage neurons [230]. The activation of AhR in brain endothelial cells weakens the barrier junction protein Occludin, enhances the permeability of the blood–brain barrier, forms a proinflammatory positive feedback pathway, and causes the continuous amplification of neuroinflammation. The disruption of the blood–brain barrier is associated with cognitive impairment diseases in humans. AhR can link the microbiota and neuroinflammation through its metabolites, especially the tryptophan metabolite, which plays an important anti‐inflammatory role in the CNS [231]. ILA from gut microbiota can reduce neuroinflammatory manifestations and inhibit the progression of amyloidosis in 5×FAD mice by activating AhR signaling in the brain [228, 229].

In addition to triggering inflammation, AhR also mediates synaptic and neural circuit dysfunction. Dysregulation of IPA/AhR/NF‐κB axis signaling in IUGR rats leads to abnormal pruning of neuronal synapses, reducing the susceptibility to autism in the offspring of intrauterine growth restriction [232]. The AhR of microglia can promote myelin regeneration in demyelinating mice and improve demyelinating diseases of the CNS [233].

AhR also plays a crucial role in the imbalance of protein homeostasis. The activation of AhR can significantly upregulate the expression and activity of neprilysin (NEP). As a direct transcription factor of NEP, AhR enhances the degradation of amyloid β and improves the cognitive impairment deficiency in mice with Alzheimer's disease [234]. These mechanisms work together through AhR in neurodegenerative diseases, highlighting the core position of AhR as a crossover node.

## 5 Therapeutic Targeting of AhR: Agonists, Antagonists, and Beyond

### 5.1 Preclinical Evidence and Mechanistic Insights

AhR functions as a pivotal transcription factor that regulates immunity, metabolism, and cell fate, and it engages in extensive cross‐talk with multiple signaling pathways within the body. This section will focus on mechanistic insights into AhR as a therapeutic target and validation evidence in related disease models.

AhR can reshape immune homeostasis through a variety of agonists in autoimmune and inflammatory diseases. The first is to regulate the differentiation of T cells, promote the differentiation of CD4^+^T cells into Treg cell, exert the immunosuppressive characteristics of Treg cell, and induce immune tolerance. The second is to inhibit multiple downstream pathways related to inflammation and control the transcriptional expression of TNF‐α, IL‐6 and other proinflammatory factors. Third, it regulates the phenotypes of a variety of innate immune cells and promotes the polarization of tolerance phenotypes of macrophages and DCs.

A large number of preclinical studies provide strong evidence. In addition to its important applications in the field of AD, tapinarof has also shown amazing therapeutic potential in preclinical studies in SLE model mice. Treatment with tapinarof, an agonist of AhR, has demonstrated efficacy in ameliorating lupus phenotypes, including splenomegaly, lymphadenopathy, renal impairment, immune complex deposition, and hypersecretion of antibodies. In MRL/lpr mice treated with tapinarof, there was a notable increase in the frequency of Treg cell subpopulations, accompanied by a reduction in the Th1/Th2 cell ratio following tapinarof administration. The study revealed that tapinarof impedes Tfh cell differentiation by modulating the JAK2–STAT3 signaling pathway and also suppresses the GC response [112]. Furthermore, tapinarof has been shown to regulate the expression of proinflammatory cytokines in activated peripheral blood CD4^+^ T cells, suggesting that it may directly influence T cell activation and cytokine production, thereby modulating the immune response. As a metabolite of dietary tryptophan, IPA has been shown to effectively ameliorate the progression of ESS and restore MDSC functionality [105]. In mouse models of EAE, the natural AhR ligand gallic acid (GA) was identified, and it was demonstrated that GA activation of AhR suppresses the production of proinflammatory cytokines IL‐6, IL‐1β, TNF‐α, and IL‐17, while enhancing the production of the anti‐inflammatory cytokine TGF‐β, thereby mitigating the severity of EAE [106]. Simultaneously, GA was observed to decrease the frequency of CD4^+^IL‐17^+^ T cells while increasing the frequency of Foxp3^+^CD4^+^ T cells.

In contrast, AhR inhibitors reversed immune tolerance against tumor progression. In the tumor microenvironment, the continuously activated AhR is a key link mediating immune escape and tumor progression. Therefore, antagonizing the function of AhR has become a new strategy for tumor treatment. Its mechanism of action includes the following parts, mainly blocking the AhR pathway and relieving the immunosuppressive effect of AhR‐activated immune cells. Or target key enzymes of the kynurenine pathway to reduce the production of endogenous AhR ligands. The existing AhR antagonists are in a large number of preclinical studies. The common antagonist CH223191 can effectively block TCDD‐mediated ERα protein degradation and CYP1A1/B1 induction, which is beneficial for ER+ breast cancer patients [235].

### 5.2 Current Clinical Trials and Translational Challenges

Despite substantial advancements in the investigation of AhR, its precise mechanistic role across various diseases remains inadequately understood and warrants further exploration. The effects of AhR activation or inhibition are highly contingent upon the specific environmental context and ligand characteristics, essentially stemming from the multidimensional plasticity of AhR signaling. This underscores the necessity for developing precise AhR regulators as a pivotal avenue for future research.

Presently, only a limited number of AhR‐targeted therapies have been approved for marketing. The vast majority of AhR modulators fail to enter the clinic due to lack of tissue selectivity or toxicity. The key issues to be resolved at present are to sort out the effects and effectiveness of AhR interventions in different disease conditions, to clarify when activation is needed, when inhibition is needed, and how to break through the biological barriers to achieve the delivery of the problem, and to reveal the principles of AhR‐targeted therapies. As shown in Table 3, the clinical and preclinical animal experiments related to AhR have been systematically summarized, which have demonstrated therapeutic efficacy in a variety of pathological models, and through the analysis of these cases, we have summarized the therapeutic modality of AhR, which is “localizable and modifiable” (Table 3).

**TABLE 3:** Clinical/preclinical animal experiments related to AhR.

Categories	Disease models	Corresponding animal/cell model	AhR intervention approach	Therapeutic effects	References
In vitro cell experiments	Tumor models	Human matricellular plasmacytoid dendritic cell tumor cell line CAL‐1	Optimizing CAR‐T therapy: CRISPR–Cas9 knocks out the AhR gene in T cells	Enhanced T‐cell activity and promotion of memory phenotype differentiation; prolonged antitumor duration and efficacy	[236]
In vitro cell experiments	Rosacea	Mast cell	Using tapinarof	Inhibition of mast cell degranulation, reduction of IL‐6/TNF‐α/MMP9 expression levels, and amelioration of inflammation	[115]
Animal experimentation	Oxidative stress liver injury	BALB/c mice	FICZ combined heavy metal exposure	Increase in GSH/GSSG ratio, metabolic disorders in hepatocytes	[237]
Animal experimentation	Traumatic brain injury	Mice	Intraperitoneal injection of 3‐n‐butylphthalide (NBP)	Attenuates neuronal iron death and inhibits microglia activation; improves motor function	[238]
Animal experimentation	Strokes	Cerebral ischemia/reperfusion injury (CIRI)	Oral isorhamnetin (ISOR)	Targeting AhR to inhibit the TLR4 signaling pathway	[238]
Animal experimentation	Heart transplant rejection	Mice	TCDD injection	Expansion of Treg cells to elevate IL‐10 levels	[239]
Animal experimentation	Breast cancer lung metastasis	Mice	Specific knockout of macrophage AhR	Reduction of pulmonary metastatic nodules; prolonged survival	[212]
Animal experimentation	Obesity	High‐fat diet mice	Feeding with AhR antagonist NF mixed diets	Preventing the onset of obesity; reversing established obesity	[240]
Clinical trial phase I	Ulcerative colitis		Oral AhR agonist Indigo naturalis (NCT02442960)	Significantly reduced inflammatory markers	[241]
Clinical trial phase III	Psoriasis		Tapinarof localized administration (NCT05014568)	Phase III trial showed >40% regression of lesions	[157, 242]
Clinical trial phase II	Solid tumor microenvironment		AhR inhibitor BAY2416964 combined with immunotherapy (NCT04069026)	Inhibition of tumor cell migration and EMT	[243]

### 5.3 Novel Strategies: Targeting the Microbiome–AhR Axis

The microbiota is recognized as a critical determinant of host health. Advancements in technology are progressively elucidating the intricate interactions between the host and the colonizing human flora [244]. AhR functions as a critical mediator in the interaction between microbial communities and their host organisms. The microbiome–AhR axis has emerged as a promising therapeutic strategy. This section will explore the mechanistic and preclinical evidence supporting the potential of targeting the microbiome–AhR axis for disease treatment.

The human gut microbiota functions as an extensive “metabolic factory”, producing a substantial array of active metabolites, many of which act as AhR agonists. Consequently, the composition and metabolic state of an individual gut microbiota significantly influence the extent of AhR signaling output. This axis was first studied in the gut, which maintains immune homeostasis through the following mechanisms. The first is to preserve the integrity of the intestinal mucosal barrier. AhR is highly expressed within the epithelial barrier, and prior research has shown that AhR knockout mice have insufficient intestinal barrier function [245]. AhR ligands generated by *Lactobacillus reuteri* activate AhR through the IL‐22, thereby modulating the secretion of AMPs and maintaining mucosal homeostasis in the gastrointestinal tract [246]. This suggests that normal expression of AhR or production of microbial‐derived AhR ligands can reduce intestinal barrier damage and enhance tolerance to inflammatory stimulation. Activation of AhR by metabolites of intestinal flora can mediate the development and differentiation of intraepithelial immune cells, reduce the production of proinflammatory cells, and regulate the overall immune environment [247].

As a systemic regulatory network, the microbiome–AhR axis not only plays a role in gut‐related diseases. The gut–brain axis has become an important area of research to reveal the complex interrelationships between microbes and the nervous system. Modifications in the composition and function of gut microbiota influence brain homeostasis, contribute to the onset of neuropsychiatric disorders, and facilitate the progression of neurodegenerative diseases [248]. For example, gut microbial metabolites are significantly different between Alzheimer's disease patients and healthy individuals [249]. The indole molecules produced by the gut microbiota are converted to the sulfate indole group in the liver, and this substance has a detrimental effect on AD. Several studies have shown that the use of probiotics intervention can improve cognitive impairment in AD patients [250]. The regulation of gut microbiota metabolism through dietary modification or probiotic therapy is regarded as an effective treatment strategy. *Lactobacillus* and *Bifidobacterium* are the most frequently utilized probiotics in therapeutic interventions.

In addition to, the microbiome–AhR axis plays a role in unexpected areas. Gut microbiota‐derived tryptophan metabolites drive disease progression in renal fibrosis [25]. These findings highlight the potential of the microbiome–AhR axis as a therapeutic target for disease intervention. However, great challenges remain and considerable variation in the composition of probiotic supplements and treatment outcomes. At present, there is no consensus on the formulation, dosage, and treatment of bacteria.

## 6 Conclusions and Outlook

As awareness of AhR continues to deepen, it has gone beyond the traditional category of “toxic pollutants”. From the current study, AhR not only plays an indispensable role in basic physiological functions such as immune regulation, cell differentiation, and metabolic homeostasis, but also exhibits a complex regulatory network in the pathogenesis of various diseases, and its bi‐directional plasticity has made it a unique therapeutic target, which subverts the single concept of traditional pharmacology.

Nevertheless, numerous critical challenges remain unresolved in the current field, impeding the translation of AhR from experimental trials to clinical application. The physiological effects of AhR ligands exhibit considerable diversity, with their actions being specific to particular cells, tissues, and species. The underlying mechanisms responsible for this selectivity remain incompletely elucidated, representing a critical area of investigation for the development of selective AhR modulators (SAhRMs) [251]. Despite the presence of the same ligand, markedly different signaling outcomes can manifest across various tissues or cells within an individual. This observation prompts an inquiry into whether such specificity is attributable to distinct cellular contexts, such as variations in receptor or cofactor expression, or to differences in protein–protein interaction networks. Understanding this concept is essential for the development of AhR‐targeted therapies specific to particular tissues. How to accurately use AhR to carry out treatment at different stages of disease development is the focus of our research.

In the future, we need to rely on some interdisciplinary advanced technologies to achieve technological innovation and breakthrough. The identification or generation of novel ligands and the investigation of their interactions with AhR represent key areas of future research. The kinetic changes associated with AhR activation have been elucidated through the application of split NanoBiT technology and cryo‐electron microscopy [252, 253]. It is not limited to the development of a single agonist or antagonist, but rather to the design of SAhRMs to maximize efficacy. The ultimate goal of the research is to construct a dynamic system that is compatible with the environment, and we will eventually witness the powerful charm of AhR‐targeted therapy in the future.

## Author Contributions

Haonan Li: Organize literature and original draft writing, search literature, and writing editing. Yufeng Fan and Jizheng Liu: Review and revision of the original draft. Yukai Jing: Conception and writing review. Huifeng Shang: Presentation of the article concept. Yunfei Zhang, Xiaocui Wang, Ying Hu, and Bin Wen: Writing and revising of the manuscript. Xuemei Duan, Ze Yan, and Shumin Dong: Checking the labelling of charts. All authors contributed to the writing and revising of the manuscript.

## Ethics Statement

The authors have nothing to report.

## Conflicts of Interest

The authors declare no conflicts of interest.

## Data Availability

The authors have nothing to report.
